# The Urethral Microbiota of Men with and without Idiopathic Urethritis

**DOI:** 10.1128/mbio.02213-22

**Published:** 2022-10-03

**Authors:** Erica L. Plummer, Larissa K. Ratten, Lenka A. Vodstrcil, Gerald L. Murray, Jennifer A. Danielewski, Christopher K. Fairley, Suzanne M. Garland, Eric P. F. Chow, Catriona S. Bradshaw

**Affiliations:** a Central Clinical School, Monash University, Melbourne, Australia; b Melbourne Sexual Health Centre, Alfred Health, Melbourne, Australia; c Melbourne School of Population and Global Health, The University of Melbourne, Melbourne, Australia; d Molecular Microbiology Research Group, Murdoch Children’s Research Institute, Melbourne, Australia; e Centre for Women’s Infectious Diseases, The Royal Women’s Hospital, Melbourne, Australia; f Department of Obstetrics and Gynaecology, The University of Melbourne, Melbourne, Australia; University of Maryland, School of Medicine

**Keywords:** idiopathic urethritis, nongonococcal urethritis, urethral microbiota, *Corynebacterium*, *Haemophilus influenzae*, sexually transmitted diseases, urethral microbiome

## Abstract

Nongonococcal urethritis (NGU) is a common genital tract syndrome in men, and up to 50% of cases are considered idiopathic, i.e., no etiological agent is identified. This poses challenges for clinicians in the diagnosis and treatment of NGU and often results in antibiotic misuse and overuse. Therefore, to identify potential infectious causes of urethritis and inform clinical management of urethritis cases, we characterized and compared the urethral microbiota of men with and without idiopathic urethritis. Participants were derived from a case-control study that examined viral and bacterial pathogens and sexual practices associated with NGU. Men with NGU who tested negative for established causes of NGU (Chlamydia trachomatis, Mycoplasma genitalium, Trichomonas vaginalis, adenoviruses, herpes simplex virus [HSV]-1, and/or HSV-2) were classified as idiopathic cases, and the controls were men reporting no current urethral symptoms. Men provided a urine sample that was used to characterize the urethral microbiota using 16S rRNA gene sequencing. Bacterial taxa associated with idiopathic urethritis were identified using analysis of compositions of microbiomes with bias correction. When stratified by sex of sexual partner, we found that the abundance of Haemophilus influenzae was significantly increased in men who have sex with men with idiopathic urethritis, and the abundance of Corynebacterium was significantly increased in men who have sex with women with idiopathic urethritis. Other taxa, including Ureaplasma, Staphylococcus haemolyticus, Streptococcus pyogenes, Escherichia, and Streptococcus pneumoniae*/*pseudopneumoniae, dominated the urethral microbiota of idiopathic urethritis cases but not controls, suggesting that these organisms may also contribute to urethritis. Importantly, the taxa we identified represent biologically plausible causes of urethritis and should be prioritized for future study.

## INTRODUCTION

Nongonococcal urethritis (NGU) is characterized by urethral inflammation in the absence of Neisseria gonorrhoeae. NGU is one of the commonest genital tract syndromes in men, and symptoms include dysuria, urethral itching and/or burning, and urethral discharge (1). Chlamydia trachomatis and Mycoplasma genitalium are the most common causes of NGU, accounting for 20 to 50% and 10 to 30% of NGU cases, respectively (2), and other less common causes include Trichomonas vaginalis, herpes simplex virus (HSV), and adenovirus. However, upwards of 50% of NGU cases are idiopathic (3–7). While some cases of idiopathic urethritis are noninfectious (6), a significant proportion are likely due to an unidentified sexually transmitted pathogen(s). Previous studies suggest that the etiology of idiopathic urethritis differs between men who have sex with men (MSM) and men who have sex with women (MSW) and/or by anatomical site of urethral exposure (3, 4, 8). Determining the infectious agents of idiopathic urethritis among MSM and MSW is important for informing both diagnostic and treatment guidelines. Molecular methods, such as 16S rRNA gene sequencing, have recently enabled comprehensive characterization of the male urinary and urethral microbiota (9–11); however, to date, there have been few studies that have compared the urethral microbiota of men with and without idiopathic urethritis (4, 12). To provide a better understanding of potential infectious causes of urethritis in men, we conducted a case-control study of men attending a sexual health service and characterized the urethral microbiota of men with and without idiopathic urethritis. To further inform clinical practice, we also investigated the association between the urethral microbiota and specific symptoms and signs of urethritis.

## RESULTS

### Participant characteristics.

**FIG 1:**
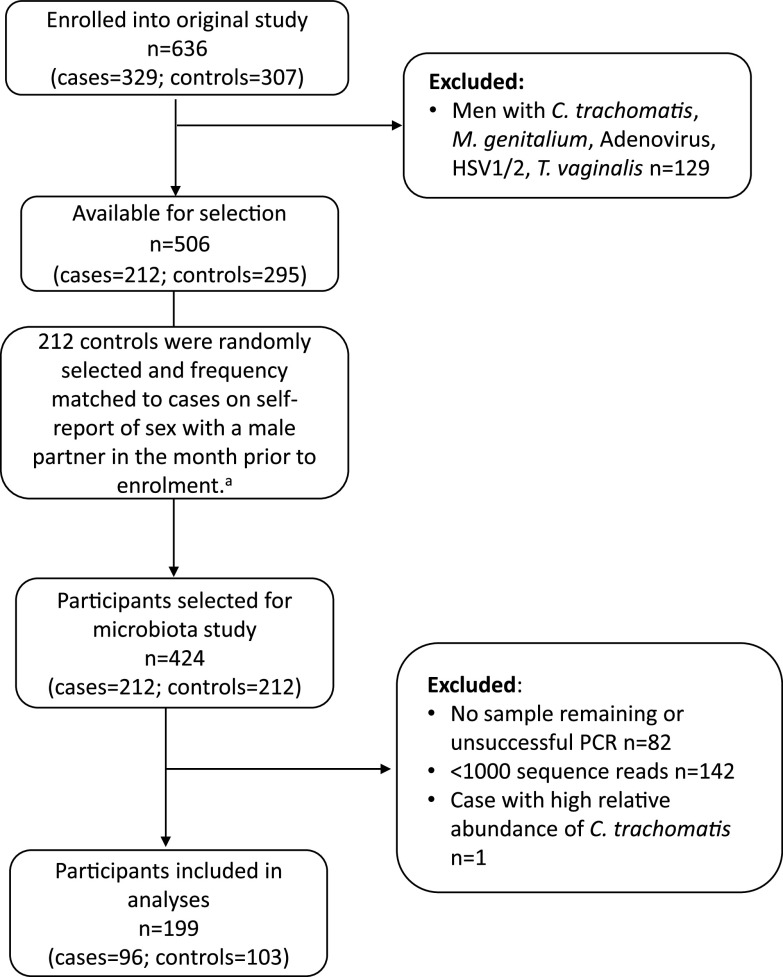
Flow chart of participants selected for inclusion in this microbiota study and the resulting number of samples included in analyses. ^a^Controls were randomly selected and frequency matched to controls to ensure a similar distribution of men who have sex with men among cases and controls.

Of the 424 urine samples eligible for inclusion, 342 (81%) had sufficient material remaining and amplified successfully. Following quality control and filtering of contaminants, 142 (42%) were excluded as they yielded <1,000 sequencing reads. One additional urine sample obtained from a case was excluded due to a high relative abundance (99.3%) of C. trachomatis (Fig. 1). As a result, sequencing data from 199 men were included in the study; this included 96 men with idiopathic urethritis and 103 controls (Fig. 1). Demographic, behavioral, and clinical characteristics were similar for men who were included and excluded, with the exception that a higher proportion of included asymptomatic controls reported a new sexual partner in the month prior to enrollment compared to excluded asymptomatic controls (57% versus 41%, chi-square *P* value 0.020).

Participant demographics, sexual practices, and clinical characteristics of the 199 men included in the final analyses are shown in Table 1. Median participant age was 31 years (interquartile range [IQR], 24–39 years) with cases being older than controls (34 years [IQR, 26–42] versus 28 years [IQR, 23–37], respectively). Seventy-one men reported a male sexual partner in the month prior to enrollment and were categorized as MSM. The remaining 128 men did not report a male sexual partner in the month prior to enrollment and were categorized as MSW. Oral sex was commonly practiced among study participants with 71 (99%) MSM and 105 (83%) MSW reporting receiving condomless oral sex during the past month. Insertive condomless anal sex in the past month was reported for 28 (39%) MSM and 19 (15%) MSW. Condomless vaginal sex in the past month was reported by 95 (74%) MSW and was more common among cases than controls (89% versus 66%, *P = *0.005). Five MSM (7%) reported a female sexual partner in the past month, and three reported condomless vaginal sex in the past month.

**TABLE 1:** Participant characteristics^*a*^

Characteristics	MSM (*N* = 71)		MSW (*N* = 128)	
	Idiopathic urethritis (*N* = 33)	Control (*N* = 38)	Idiopathic urethritis (*N* = 63)	Control (*N* = 65)
Age, median (IQR)	33 (27, 41)	28 (23, 39)	36 (26, 42)	28 (24, 34)^*b*^
Circumcised				
No	18 (55)	19 (50)	31 (49)	37 (57)
Yes	15 (45)	19 (50)	32 (51)	26 (40)
Not recorded	0 (0)	0 (0)	0 (0)	2 (3)
Sexual practices in past month				
New sexual partner				
No	4 (12)	10 (26)	26 (41)	34 (52)
Yes	29 (88)	28 (74)	37 (59)	31 (48)
Regular sexual partner				
No	19 (58)	19 (50)	23 (37)	32 (49)
Yes	14 (42)	19 (50)	40 (63)	33 (51)
≥1 Female sexual partner				
No	30 (91)	36 (95)	2 (3)^*c*^	9 (14)^*c*^
Yes	3 (9)	2 (5)	61 (97)	56 (86)^*d*^
≥ 1 Male sexual partner				
No	0 (0)	0 (0)	63 (100)	65 (100)
Yes	33 (100)	38 (100)	0 (0)	0 (0)
No. of sexual partners, median (IQR)	3 (2, 9)	3 (1, 6)	1 (1, 2)	2 (1, 2)
Received condomless oral sex				
No	1 (3)	0 (0)	12 (19)	9 (14)
Yes	32 (97)	38 (100)	50 (81)	55 (86)
Any condomless vaginal sex				
No	31 (94)	37 (97)	7 (11)	21 (34)
Yes	2 (6)	1 (3)	54 (89)	41 (66)^*b*^
Any insertive condomless anal sex				
No	22 (67)	21 (55)	52 (84)	54 (86)
Yes	11 (33)	17 (45)	10 (16)	9 (14)
Symptoms and clinical characteristics				
Dysuria				
No	10 (30)	38 (100)	22 (35)	65 (100)
Yes	23 (70)	0 (0)	41 (65)	0 (0)
Burning				
No	10 (30)	38 (100)	23 (37)	65 (100)
Yes	23 (70)	0 (0)	40 (63)	0 (0)
Self-reported urethral discharge				
No	10 (30)	38 (100)	29 (46)	65 (100)
Yes	23 (70)	0 (0)	34 (54)	0 (0)
Urethral discharge on examination				
No	14 (42)	38 (100)	35 (56)	65 (100)
Yes	19 (58)	0 (0)	28 (44)	0 (0)
Nature of urethral discharge				
None/normal	14 (42)	38 (100)	35 (56)	65 (100)
Mucoid	14 (42)	0 (0)	25 (40)	0 (0)
Mucopurulent	5 (15)	0 (0)	3 (5)	0 (0)
Meatitis^*e*^				
No	17 (52)	38 (100)	45 (71)	63 (98)
Yes	16 (48)	0 (0)	18 (29)	1 (2)
Balanitis^*e*^				
No	32 (97)	37 (97)	58 (92)	61 (95)
Yes	1 (3)	1 (3)	5 (8)	3 (5)
Epididymitis^*f*^				
No	33 (100)	37 (100)	58 (92)	63 (98)
Yes	0 (0)	0 (0)	5 (8)	1 (2)
			
>5 PMNLs/HPF on urethral Gramstain^*g*^				
No	19 (58)	NA	45 (71)	NA
Yes	14 (42)	NA	18 (29)	NA

a The data are missing for up to *n* = 5 participants. HPF, high-power field; IQR, interquartile range; MSM, men who have sex with men; MSW, men who have sex with women; NA, not applicable; PMNL, polymorphonuclear leukocyte.

b *P < *0.05.

c Eleven men reported no sex in the month prior to enrollment but reported a lifetime history of only female sexual partners.

d *P* = 0.06.

e Meatitis and balanitis were not recorded for one control.

f Epididymitis was not recorded for one man with idiopathic urethritis and one control.

g Control men were not assessed for urethral polymorphonuclear leukocytes.

### Urethral microbiota of men with and without idiopathic urethritis.

**FIG 2:**
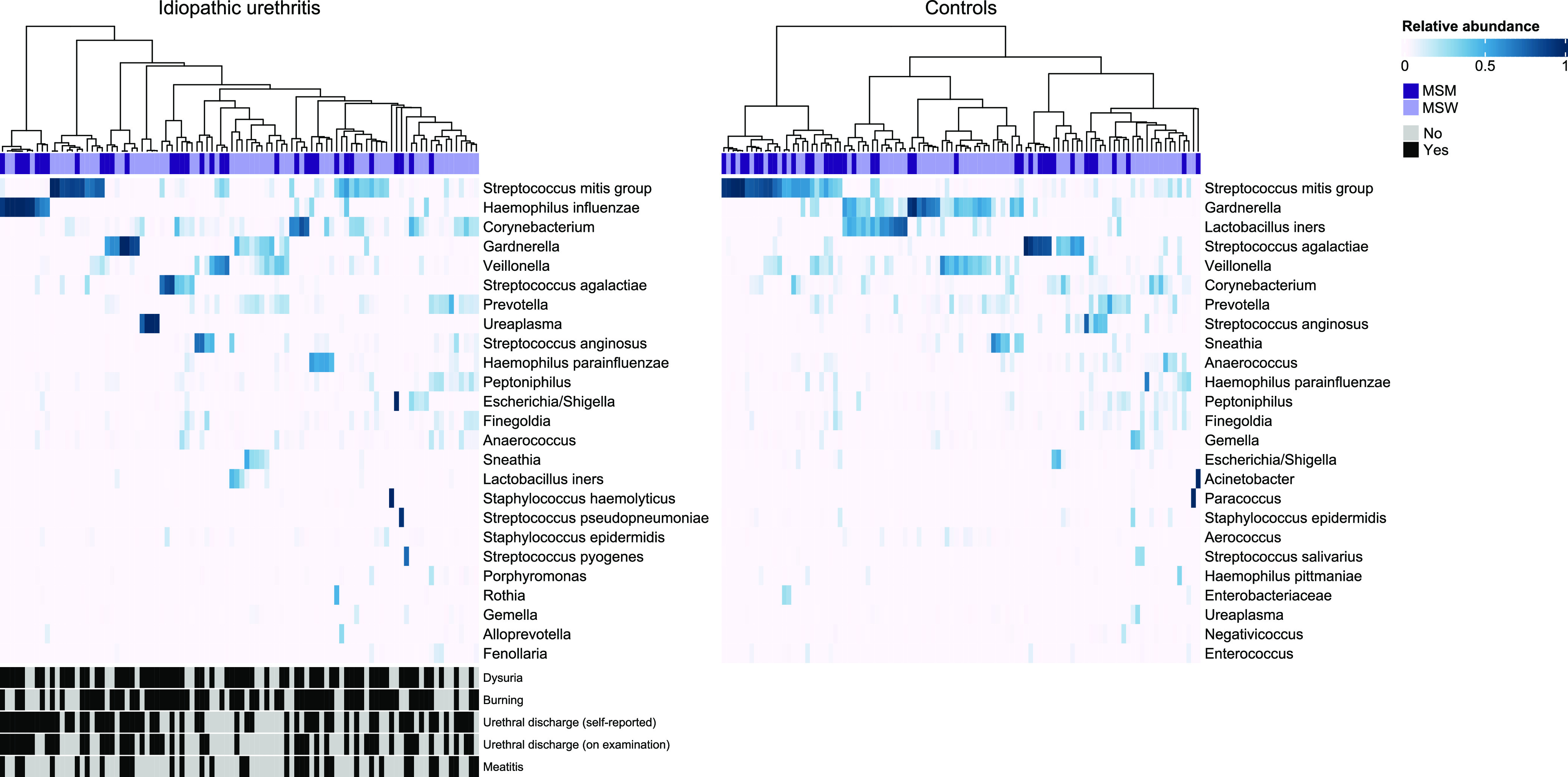
The heat map shows the relative abundance of the 25 most abundant taxa detected in the urethral microbiota of men with and without idiopathic urethritis. The metadata above the heat map indicates men who reported a male sexual partner in the month prior to enrollment (MSM) and men who did not report sex with a male partner (MSW). The presence or absence of specific self-reported urethral symptoms and clinical signs of urethritis are displayed in the metadata below the heat map.

The urethral microbiota of cases and controls is shown in Fig. 2. Several taxa were commonly detected in men with and without idiopathic urethritis, including Streptococcus mitis group (i.e., amplicon sequence variants [ASVs] matching S. mitis, Streptococcus oralis, and Streptococcus infantis), Corynebacterium, Gardnerella, Veillonella, Streptococcus agalactiae, and Prevotella. Hierarchical clustering revealed no distinct clustering according to case status. However, Haemophilus influenzae was commonly present in men with idiopathic urethritis (Fig. S1); 15 of 33 (45%) MSM with idiopathic urethritis had H. influenzae detected compared to 10 of 63 (16%) MSW with idiopathic urethritis (Fisher’s exact test *P* = 0.003). In contrast, Lactobacillus iners was common among controls, particularly MSW.

10.1128/mbio.02213-22.9 FIG S1 The heat map shows the relative abundance of the 25 most abundant taxa detected in men who reported sex with a male sexual partner in the month prior to enrollment (MSM) and men who did not report sex with a male partner (MSW). The metadata above the heat map indicates men with idiopathic urethritis (IU) and asymptomatic controls. Download FIG S1, PDF file, 0.2 MB. Copyright © 2022 Plummer et al. 2022 Plummer et al. https://creativecommons.org/licenses/by/4.0/ This content is distributed under the terms of the Creative Commons Attribution 4.0 International license.

Of note, we identified seven bacterial taxa that dominated (i.e., defined as ≥70% relative abundance) the urethral microbiota of one or more cases and did not dominate any individual in the controls. These taxa included H. influenzae (five MSM, three MSW), Ureaplasma (four MSW), Staphylococcus haemolyticus (one MSW), Streptococcus pyogenes (one MSW), and Escherichia/Shigella, likely representing Escherichia coli (one MSM). Additionally, one MSM was dominated by a single ASV that had high identity (99.7%) to both Streptococcus pneumoniae and Streptococcus pseudopneumoniae. Corynebacterium was common in both cases and controls but dominated only the urethral microbiota of cases (one MSM and one MSW). Gardnerella S. mitis group, S. agalactiae, and Streptococcus anginosus dominant communities were identified in both cases and controls. L. iners dominant communities were present only in controls (*n* = 3, all MSW).

We identified a small but significant difference in the global composition of the urethral microbiota between cases and controls (analysis of similarity [ANOSIM] R statistic = 0.04, *P = *0.001; Fig. S2A), and between MSM and MSW (ANOSIM R statistic = 0.06, *P = *0.003; Fig. S2B). Similarly, analyses stratified by MSM status identified a significant difference in the overall composition of the urethral microbiota of cases compared to controls among both MSM (ANOSIM R statistic = 0.07, *P = *0.007; Fig. S2C) and MSW (ANOSIM R statistic = 0.04, *P = *0.002; Fig. S2D). There was no significant difference in the bacterial diversity between cases and controls among either MSM or MSW (Fig. S2E).

10.1128/mbio.02213-22.10 FIG S2 (A to D) Nonmetric multidimensional scaling (NMDS) plots of the male urethral microbiota. Individual dots represent a single urine sample, and the dots have been colored according to relevant metadata. 95% confidence ellipse plots have been included. Analysis of similarity (ANOSIM) test statistics are shown in the top left corner of each plot, and *P < *0.05 indicates dissimilarity in the global composition of the urethral microbiota between men with and without idiopathic urethritis (A); men who reported sex with a male partner in the month prior to enrollment (MSM) and men who did not report sex with a male partner (MSW) (B); men with and without idiopathic urethritis among MSM (C); and men with and without idiopathic urethritis among MSW (D). (E) Box plots showing the bacterial diversity (measured using the Shannon diversity index) of the urethral microbiota of men with and without idiopathic urethritis (IU). There was no significant difference in the bacterial diversity between men with idiopathic urethritis and controls among either men who have sex with men (MSM; coefficient = 0.03; 95% CI = −0.37, −0.44; *P* = 0.872) or MSW (coefficient = −0.08; 95% CI = −0.38, −0.22; *P* = 0.603). Download FIG S2, PDF file, 0.2 MB. Copyright © 2022 Plummer et al. 2022 Plummer et al. https://creativecommons.org/licenses/by/4.0/ This content is distributed under the terms of the Creative Commons Attribution 4.0 International license.

In analysis of compositions of microbiomes with bias correction (ANCOM-BC) analyses of the whole study population, the mean abundance of both H. influenzae and Corynebacterium was significantly higher in men with idiopathic urethritis compared to controls (coefficient = 1.21, false discovery rate [FDR] *P = *0.030 and coefficient = 0.79, FDR *P = *0.088, respectively; Table 2). Conversely, Gardnerella, L. iners, Aerococcus, and Gemella had a significantly higher mean abundance in control men (coefficients ranged from −1.41 to −0.73, FDR *P < *0.10).

**TABLE 2:** Differentially abundant organisms between men with and without idiopathic urethritis^*a*^

Analysis	Idiopathic urethritis (*n* [%])^*b*^	Control (*n* [%])^*b*^	Coefficient^*c*^	SE	*P* value	FDR-adjusted *P* value^*d*^
Unstratified analysis	*N* = 96	*N* = 103				
Haemophilus influenzae	25 (26)	14 (14)	1.21	0.38	0.002	**0.030**
Corynebacterium	82 (85)	89 (86)	0.79	0.31	0.012	**0.088**
Gemella	26 (27)	49 (48)	−0.73	0.29	0.012	**0.088**
Aerococcus	13 (14)	28 (27)	−0.81	0.26	0.002	**0.021**
Lactobacillus iners	13 (14)	34 (33)	−1.36	0.39	<0.001	**0.021**
Gardnerella	31 (32)	49 (48)	−1.41	0.50	0.005	**0.054**
Stratified analysis						
MSM	*N* = 33	*N* = 38				
Haemophilus influenzae	15 (45)	4 (11)	3.39	0.89	<0.001	**0.004**
Porphyromonas	8 (24)	7 (18)	1.10	0.53	0.039	0.248
Haemophilus pittmaniae	2 (6)	8 (21)	−0.84	0.37	0.022	0.231
Gardnerella	6 (18)	14 (37)	−1.62	0.77	0.034	0.248
Streptococcus mitis group	23 (70)	34 (89)	−2.02	0.72	0.005	**0.084**
MSW	*N* = 63	*N* = 65				
Corynebacterium	57 (90)	55 (85)	1.05	0.37	0.005	**0.055**
Aerococcus	11 (17)	23 (35)	−1.11	0.37	0.003	**0.055**
Gemella	17 (27)	35 (54)	−1.13	0.39	0.003	**0.055**
Lactobacillus iners	13 (21)	27 (42)	−1.57	0.56	0.005	**0.055**
Gardnerella	25 (40)	35 (54)	−1.58	0.65	0.015	0.133

a FDR, false discovery rate; MSM, men who have sex with men; MSW, men who have sex with women; SE, standard error.

b *n* = number of men with the specific taxon detected, % = n/N, i.e., the percentage of cases (or controls) with the specific taxon detected.

c Coefficients were obtained from the analysis of compositions of microbiomes with bias correction (ANCOM-BC) log-linear (natural log) model. Positive coefficients indicate higher abundance in men with idiopathic urethritis, whereas negative coefficients indicate a higher abundance in control men. Analyses were adjusted for age and sequencing run, and only taxa with *P* values less than 0.05 are included in this table.

d Bold type indicates that the difference was considered statistically significant (*P*, 0.05, FDR *P*, 0.1).

**FIG 3:**
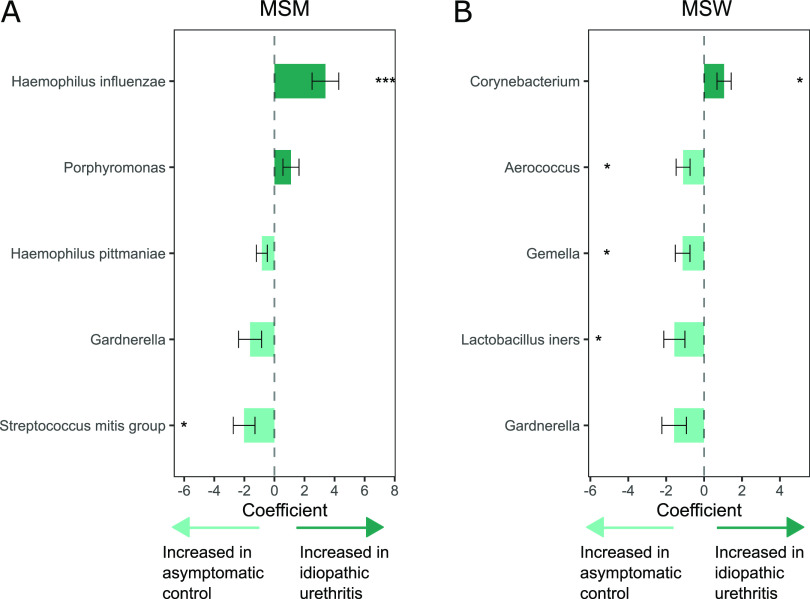
Bacterial taxa identified as differentially abundant between men with idiopathic urethritis and asymptomatic controls among MSM (A) and MSW (B). The colored bars represent the coefficients obtained from the analysis of compositions of microbiomes with bias correction (ANCOM-BC) log-linear (natural log) model, and the error bars show the standard error around the estimate. Positive coefficients (dark green) indicate higher abundance in men with idiopathic urethritis, whereas negative coefficients (light green) indicate a higher abundance in controls. All taxa with *P* < 0.05 are shown in the figure. *, false discovery rate (FDR)-adjusted *P* < 0.1; ***, FDR-adjusted *P* < 0.01.

In ANCOM-BC analyses stratified by gender of sexual partner, the mean abundance of H. influenzae was significantly increased in MSM with idiopathic urethritis compared to controls after correcting for multiple comparisons (coefficient = 3.39, FDR *P = *0.004; Table 2; Fig. 3A). A sensitivity analysis that excluded the five MSM who reported both male and female sexual partners in the month prior to enrollment yielded similar results (Table S2). Among MSW, the mean abundance of Corynebacterium (coefficient = 1.05, FDR *P = *0.055) was significantly higher in men with idiopathic urethritis compared to controls (Table 2; Fig. 3B). Conversely, S. mitis group had a significantly higher mean abundance in controls versus cases among MSM (coefficient = −2.02, FDR *P = *0.084; Table 2; Fig. 3A), and L. iners, Gemella, and Aerococcus had a higher mean abundance in controls versus cases among MSW (coefficients ranged from −1.57 to −1.11, FDR *P* < 0.10; Table 2; Fig. 3B).

10.1128/mbio.02213-22.2 TABLE S2 Sensitivity analysis exploring differentially abundant organisms between men who have sex with men (MSM) with and without idiopathic urethritis. Download Table S2, DOCX file, 0.01 MB. Copyright © 2022 Plummer et al. 2022 Plummer et al. https://creativecommons.org/licenses/by/4.0/ This content is distributed under the terms of the Creative Commons Attribution 4.0 International license.

### Urethral microbiota composition and clinical characteristics of urethritis.

**FIG 4:**
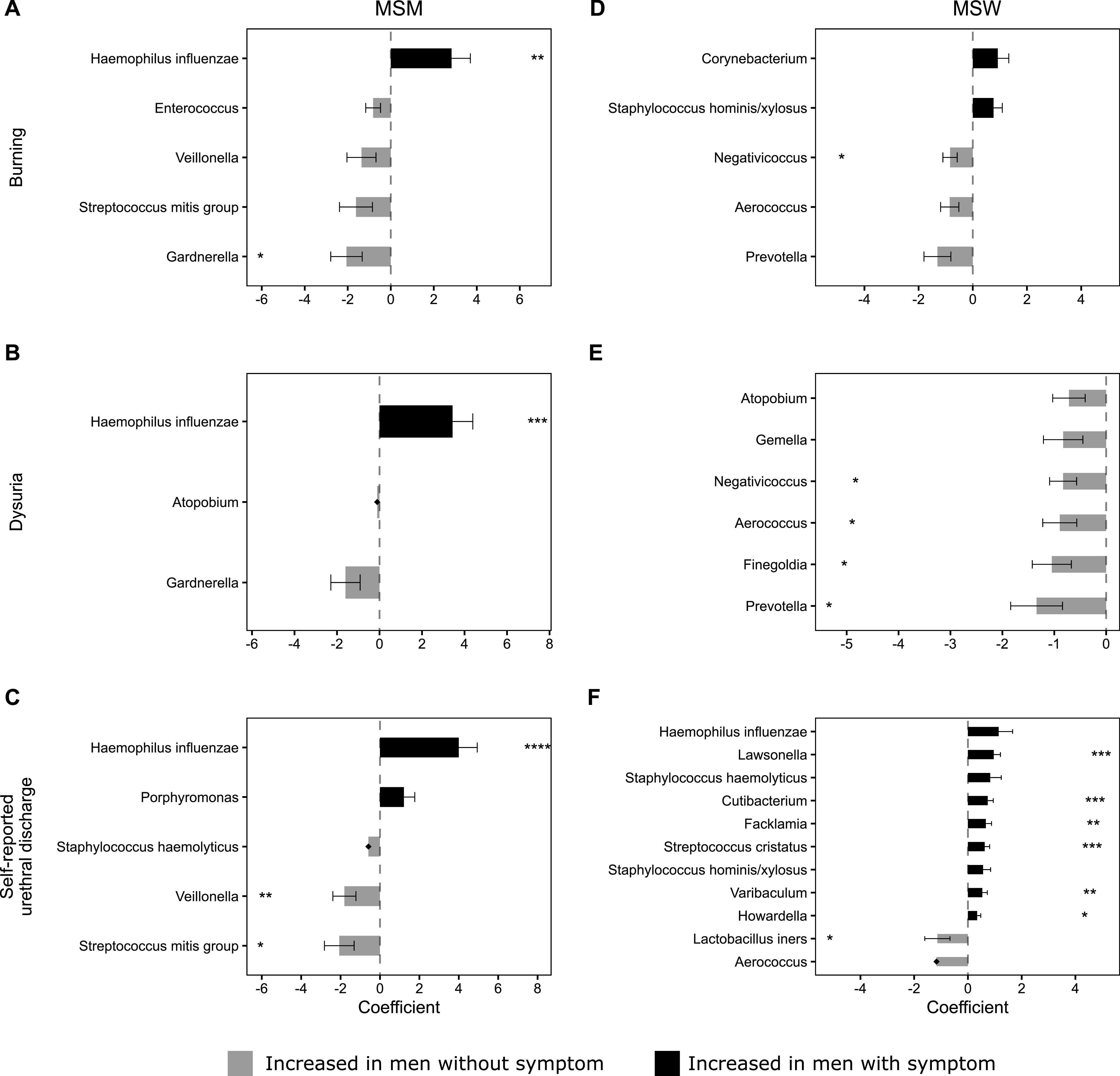
Bacterial taxa identified as differentially abundant between men with and without urethral symptoms. (A) MSM with urethral burning versus MSM without burning. (B) MSM with dysuria versus MSM without dysuria. (C) MSM with self-reported urethral discharge versus MSM without self-reported discharge. (D) MSW with urethral burning versus MSW without burning. (E) MSW with dysuria versus MSW without dysuria. (F) MSW with self-reported urethral discharge versus MSW without self-reported discharge. The horizontal bars represent coefficients obtained from the ANCOM-BC log-linear (natural log) model. Positive coefficients (black) indicate higher abundance in men with the symptom, whereas negative coefficients (gray) indicate a higher abundance in men without the symptom. The error bars show the standard error around the estimate. Structural zeros (taxa present in one group but absent, or close to absent, from the comparator) do not have error bars. All taxa with *P* < 0.05 are shown in the figure. *, FDR-adjusted *P* < 0.1; **, FDR-adjusted *P* < 0.05; ***, FDR-adjusted, *P* < 0.01; ****, FDR-adjusted *P* < 0.001.

Fig. 4 shows the bacterial taxa that were differentially abundant between men who reported specific urethral symptoms and men who did not. After correction for multiple comparisons, the mean abundance of H. influenzae was significantly higher in MSM with urethral burning (coefficient = 2.83, FDR *P = *0.039; Table S3; Fig. 4A), dysuria (coefficient = 3.44, FDR *P = *0.010; Table S4; Fig. 4B), and self-reported urethral discharge (coefficient = 3.39, FDR *P < *0.001; Table S5; Fig. 4C) compared to men who did not report these symptoms. In contrast, Gardnerella, S. mitis group, Veillonella, and Enterococcus were increased in MSM without these specific urethral symptoms. Following FDR correction, Gardnerella was significantly increased among MSM without self-reported urethral burning (coefficient = −2.06, FDR *P = *0.079; Table S3; Fig. 4A), and both Veillonella and S. mitis group were significantly increased among MSM without self-reported urethral discharge (coefficient = −1.81, FDR *P = *0.033 versus coefficient = −2.07, FDR *P = *0.066, respectively; Table S5; Fig. 4C).

10.1128/mbio.02213-22.3 TABLE S3 Association of individual taxa with urethral burning. Download Table S3, DOCX file, 0.02 MB. Copyright © 2022 Plummer et al. 2022 Plummer et al. https://creativecommons.org/licenses/by/4.0/ This content is distributed under the terms of the Creative Commons Attribution 4.0 International license.

10.1128/mbio.02213-22.4 TABLE S4 Association of individual taxa with dysuria. Download Table S4, DOCX file, 0.02 MB. Copyright © 2022 Plummer et al. 2022 Plummer et al. https://creativecommons.org/licenses/by/4.0/ This content is distributed under the terms of the Creative Commons Attribution 4.0 International license.

10.1128/mbio.02213-22.5 TABLE S5 Association of individual taxa with self-reported urethral discharge. Download Table S5, DOCX file, 0.02 MB. Copyright © 2022 Plummer et al. 2022 Plummer et al. https://creativecommons.org/licenses/by/4.0/ This content is distributed under the terms of the Creative Commons Attribution 4.0 International license.

After correction for multiple comparisons, no taxa were significantly increased in MSW who reported urethral burning (Table S3; Fig. 4D) or dysuria (Table S4; Fig. 4E) compared to MSW who did not report these symptoms. The mean abundance of Lawsonella, Cutibacterium, Facklamia, Streptococcus cristatus, Varibaculum, and Howardella was significantly higher in MSW with self-reported urethral discharge compared to those without (coefficients ranged from 0.34 to 0.96; Table S5; Fig. 4F). However, these taxa had a low maximum relative abundance in MSW with self-reported urethral discharge (range = 0.4 to 1.9%), and some were uncommonly detected (Howardella, Varibaculum, and Cutibacterium were detected in ≤3 MSW with self-reported urethral discharge). Therefore, the clinical relevance of these bacteria is unclear. Of note, H. influenzae was also elevated in MSW with self-reported discharge (coefficient = 1.14), but this was not significant after FDR correction. Negativococcus, Aerococcus, Prevotella, Atopobium, Finegoldia, Gemella, and L. iners were increased in MSW who did not report urethral symptoms; however, not all observations remained significant following FDR correction, which is likely a result of the small sample size.

**FIG 5:**
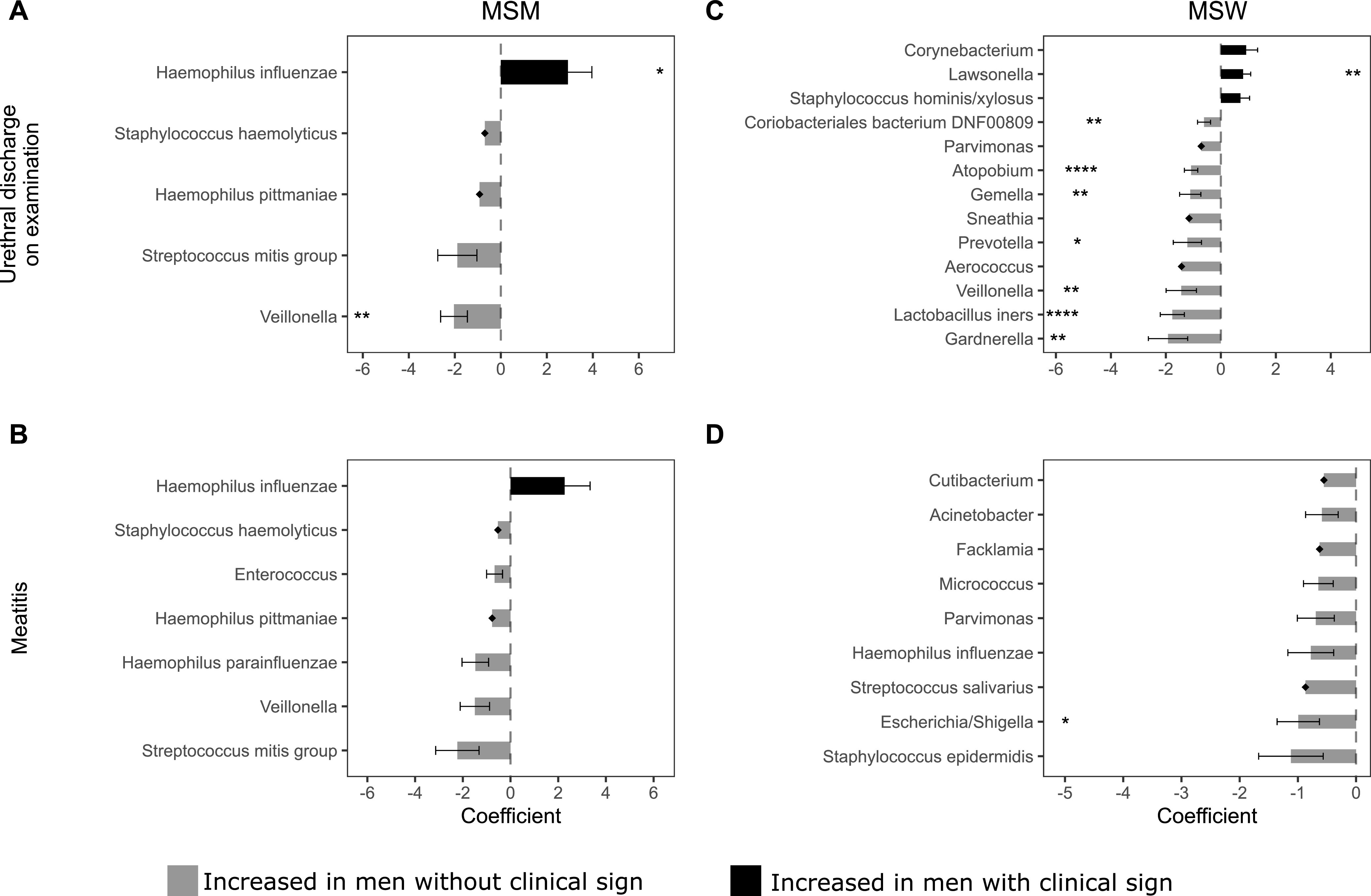
Bacterial taxa identified as differentially abundant between men with and without clinical signs of urethritis. (A) MSM with urethral discharge on examination versus MSM without urethral discharge on examination. (B) MSM with meatitis versus MSM without meatitis. (C) MSW with urethral discharge on examination versus MSW without urethral discharge on examination. (D) MSW with meatitis versus MSW without meatitis; The horizontal bars represent coefficients obtained from the ANCOM-BC log-linear (natural log) model. Positive coefficients (black) indicate higher abundance in men with the clinical sign, whereas negative coefficients (gray) indicate a higher abundance in men without the clinical sign. The error bars show the standard error around the estimate. Structural zeros (taxa present in one group but absent, or close to absent, from the comparator) do not have error bars. All taxa with *P* < 0.05 are shown in the figure. *, FDR-adjusted *P* < 0.1; **, FDR-adjusted *P* < 0.05; ***, FDR-adjusted *P* < 0.01; ****, FDR-adjusted *P* < 0.001.

Fig. 5 shows the bacterial taxa that were differentially abundant between men with and without specific clinical signs of urethritis. Among MSM, the mean abundance of H. influenzae was significantly increased in men with urethral discharge on examination compared to men without discharge (coefficient = 2.92, FDR *P = *0.065; Table S6; Fig. 5A). Conversely, both S. mitis group and Veillonella were increased in MSM without urethral discharge; only Veillonella remained significantly differentially abundant after correction for multiple comparisons (coefficient = −2.04, FDR *P = *0.015). Following FDR correction, no taxa were significantly differentially abundant in MSM with meatitis (Table S7; Fig. 5B).

10.1128/mbio.02213-22.6 TABLE S6 Association of individual taxa with urethral discharge on examination. Download Table S6, DOCX file, 0.02 MB. Copyright © 2022 Plummer et al. 2022 Plummer et al. https://creativecommons.org/licenses/by/4.0/ This content is distributed under the terms of the Creative Commons Attribution 4.0 International license.

10.1128/mbio.02213-22.7 TABLE S7 Association of individual taxa with meatitis. Download Table S7, DOCX file, 0.02 MB. Copyright © 2022 Plummer et al. 2022 Plummer et al. https://creativecommons.org/licenses/by/4.0/ This content is distributed under the terms of the Creative Commons Attribution 4.0 International license.

After correction for multiple comparisons, the mean abundance of Lawsonella was significantly higher in MSW with urethral discharge on examination compared to those without discharge on examination (coefficient = 0.81, FDR *P* value = 0.025; Table S6; Fig. 5C). In contrast, the mean abundance of Coriobacteriales bacterium DNF00809, Atopobium, Gemella, Prevotella, Veillonella, L. iners, and Gardnerella was significantly higher in MSW without discharge on examination (coefficients ranged from −0.61 to −1.92, FDR *P < *0.10). Following FDR correction, Escherichia/Shigella was increased in MSW without meatitis (coefficient = −0.99, FDR *P* value = 0.095); no other taxa were differentially abundant between MSW with and without meatitis (Table S7; Fig. 5D).

### Urethral microbiota composition and sexual practices.

We next investigated differences in the taxonomic composition of the urethral microbiota by anatomical site of urethral exposure during sex (Table S8). After correction for multiple comparisons, the mean abundance of six bacterial taxa (L. iners, Prevotella, Ureaplasma, Atopobium, Aerococcus, and C. bacterium DNF00809) were significantly higher in men reporting condomless vaginal sex compared to men who did not report condomless vaginal sex (coefficients ranged from −1.25 to 0.68, FDR *P < *0.05). Following FDR correction, no taxa were significantly differentially abundant between men reporting receptive oral sex and men not reporting the practice or between men reporting insertive anal sex and men not reporting the practice.

10.1128/mbio.02213-22.8 TABLE S8 Association of individual taxa with sexual exposure. Download Table S8, DOCX file, 0.02 MB. Copyright © 2022 Plummer et al. 2022 Plummer et al. https://creativecommons.org/licenses/by/4.0/ This content is distributed under the terms of the Creative Commons Attribution 4.0 International license.

## DISCUSSION

In this case-control study, we found that the overall composition of the urethral microbiota differed between men with and without idiopathic urethritis and differed by sex of sexual partner. We identified key bacterial taxa that were associated with idiopathic urethritis. In stratified analyses, we found that the abundance of H. influenzae was increased in MSM with idiopathic urethritis, and the abundance of Corynebacterium was higher in MSW with idiopathic urethritis. In addition, Ureaplasma spp., S. haemolyticus, S. pyogenes, Escherichia, and S. pneumoniae*/*pseudopneumoniae dominated the urethral microbiota of some men with idiopathic urethritis but not controls, indicating that these taxa may represent uncommon infectious causes of idiopathic urethritis. Together, these findings suggest that a range of bacteria may cause idiopathic urethritis in men and that these pathogens may differ according to the sex of sexual partner and/or anatomical site of urethral exposure during sex.

In addition to the finding that H. influenzae was more abundant in MSM with idiopathic urethritis compared to controls, we found that among MSM, H. influenzae was positively associated with each of the five clinical characteristics of urethritis that were recorded in the parent study. Furthermore, H. influenzae abundance was higher in MSW with self-reported urethral discharge compared to those without discharge, and three MSW had a urethral microbiota dominated by H. influenzae. Consistent with previous molecular and culture-based studies and case reports (4, 13–16), these data suggest that H. influenzae is a likely cause of idiopathic urethritis and urethral symptoms in men. H. influenzae is present in the nasopharynx of >75% of healthy adults (17); therefore, the most plausible route of transmission is via condomless oral sex (13). There is now considerable epidemiological and behavioral data that are entirely consistent with this transmission route: 99% of MSM and 83% of MSW in our study reported receiving condomless oral sex in the month prior to enrollment, and oral sex is an independent risk factor for pathogen-negative NGU (3). H. influenzae was present in 26% of men with idiopathic urethritis in our study and was significantly more common in MSM with idiopathic urethritis compared to MSW with idiopathic urethritis (45 and 16% of cases, respectively; *P* = 0.003), which may reflect the higher partner numbers among MSM compared to MSW.

Other studies of men attending sexual health services have reported similar prevalence of H. influenzae. For example, a North American study noted that 27% of men with idiopathic urethritis had H. influenzae detected by quantitative PCR (4), and a study of Japanese men reported H. influenzae was detected in 14% men with nonchlamydial NGU (15). Together, these data suggest that H. influenzae may be a common cause of urethritis in some populations. The observation that the first line treatment for NGU (doxycycline) is effective for idiopathic urethritis (18) is also consistent with H. influenzae being a common cause.

Corynebacterium was commonly detected in men with and without idiopathic urethritis. Corynebacterium spp. are considered major constituents of the cutaneous penile microbiota and are also frequently recovered from male urine and urethral samples (4, 9, 10, 12, 19–21), as well as from vaginal samples (19, 22). There are limited data exploring what constitutes optimal genital microbiota in men; however, Corynebacterium are generally considered commensals of the male genital microbiota (23) and have been associated with positive health outcomes; Corynebacterium pyruviciproducens was negatively associated with urethritis among MSW in one study (4), and both the presence and abundance of penile Corynebacterium has been associated with optimal vaginal microbiota in female sexual partners (20). As such, the finding of increased abundance of Corynebacterium among MSW with idiopathic urethritis compared to controls is somewhat surprising. Corynebacterium is a diverse genus, and short fragments of the 16S rRNA gene are not variable enough to confidently differentiate between all species (24); therefore, it is possible that specific Corynebacterium spp. account for a small number of urethritis cases, perhaps in the setting of a high abundance/high-load infection. In support of this, there have been case reports associating individual Corynebacterium spp. with urethritis, in particular Corynebacterium glucuronolyticum (25–27) and Corynebacterium propinquum (28). The role of Corynebacterium spp. in male sexual health and urethritis requires further investigation in a larger study, and future studies should use an alternative methodology that enables species or strain level identification.

S. pneumoniae*/*pseudopneumoniae, S. pyogenes, E. coli, S. haemolyticus, and Ureaplasma were each present as a dominant community in a small number of men with idiopathic urethritis. Although some of these organisms have previously been associated with urethritis in case reports (29–36), a causative role has not been proven. If these organisms do have a causal role in idiopathic urethritis, it is likely that they account for only a small proportion of cases. Of note, Ureaplasma urealyticum and Ureaplasma parvum are both commonly recovered from the urogenital tract of sexually active men and women and have been detected in men with and without NGU (4–6, 37, 38). The hypervariable region targeted in our study is unable to distinguish U. urealyticum and U. parvum; however, in the parent study (3), neither species was associated with NGU by PCR. Similarly, using PCR, Srinivasan et al. (4) found that neither the load nor the presence of U. urealyticum was associated with NGU in their case-control study. In contrast, some studies have reported significantly higher bacterial load of Ureaplasma species, particularly U. urealyticum, among men with urethritis compared to controls (5, 37, 38), suggesting that this organism may account for a small number of urethritis cases but only in the setting of a high-load infection (39). An alternative hypothesis is that initial exposure to U. urealyticum may trigger urethritis symptoms, whereas repeat or prolonged exposure may elicit an attenuated immune response and asymptomatic infection (39, 40).

Gardnerella was commonly detected in our study; almost half of MSW and, interestingly, 28% of MSM (20 of 71), had Gardnerella present in their urethral microbiota. Of note, none of the 20 MSM with detectable Gardnerella reported a female sexual partner in the 3 months prior to enrollment, and only five reported lifetime female sexual partners. Gardnerella spp. are commonly present in the vagina, and specific Gardnerella spp. are thought to play a key role in bacterial vaginosis (BV) pathogenesis (41). An early study reported that male partners of women with BV were more likely to have NGU compared to male partners of women without BV (42), and other studies have linked individual BV-associated bacteria to NGU in heterosexual men (43, 44), suggesting a possible link between BV-associated bacteria and NGU. However, in our study, we found that the mean abundance of Gardnerella was higher in asymptomatic controls compared to cases. In addition, bacterial genera commonly associated with BV, including Prevotella, Gemella, Atopobium, and Aerococcus, were present in higher mean abundance in MSW without symptoms/clinical signs of urethritis compared to MSW with symptoms/signs of urethritis. We also observed higher abundance of L. iners, a prevalent vaginal bacterium (45), among asymptomatic MSW. Additionally, in the current study, we found that the mean abundances of L. iners, Prevotella, Ureaplasma, Atopobium, Aerococcus, and C. bacterium DNF00809 were higher in men who practiced condomless vaginal sex in the month prior to enrollment compared to those who did not. Together, these data are consistent with the parent study (3), which reported that Gardnerella vaginalis detection by PCR was more common in controls compared to men with NGU and that controls with G. vaginalis were more likely to have condomless vaginal sex in the previous 14 days compared to controls without G. vaginalis. Thus, it is highly likely that the presence of L. iners and BV-associated bacteria in the urethral microbiota of men reflects recent vaginal exposure. Interestingly, BV-associated bacteria have been detected in the genital microbiota of male partners of women without BV (10, 20), and these organisms were also present in MSM in our study without any reported exposure to women in the prior 3 months, albeit at lower frequency. Therefore, it is also possible that BV-associated bacteria are not exclusively acquired from the vagina but may form part of the indigenous male urethral microbiota or alternatively be present in the rectum or mouth. Importantly, different Gardnerella spp. are hypothesized to have different pathogenic potential in women (41); therefore, it is also possible that some Gardnerella spp. contribute to urethritis, whereas others are commensal. Larger studies investigating the male genital microbiome with greater taxonomic resolution and accompanying data on sexual practices are needed to understand the role of these organisms in men. While we found differences in the urethral microbiota of men who reported penile-vaginal exposure and those who did not, we found no difference in the abundance of any bacterial taxa according to orogenital or anogenital exposure. Paired genital specimens from sexual partners would provide important additional information about the exchange of genital microbiota between couples and the impact of sexual practices on the urethral microbiome.

There are limitations to this study. First, approximately half of the urine samples selected for inclusion in this study did not generate adequate sequencing data, due to unsuccessful PCR or a low number of sequences following quality filtering. Additionally, some samples did not have adequate sample remaining. Urine has low microbial biomass (46), and previous studies of the urinary microbiota have also reported high proportions of samples failing to generate adequate sequence data (4, 47, 48). Additionally, the parent study from which the samples were obtained was completed in 2006 (3). Although urine samples were stored at −80°C, it is possible that the length of storage negatively affected sample quality. The truncated sample size and reduced statistical power greatly affected our ability to identify significant differentially abundant organisms. Second, a higher proportion of controls included in final analyses reported recent exposure to a new sexual partner compared to excluded controls. Although it is unlikely that this significantly biased the findings, it is possible that included controls were of slightly higher risk than excluded controls, which may have also reduced our ability to discriminate between the microbiota of cases and controls. Third, participants were recruited from a single sexual health clinic, which may limit the generalizability of our findings. Fourth, the two prior studies that investigated the urethral microbiome of men with urethritis used alternative definitions for urethritis cases and controls (4, 12), which may limit comparability across studies. Furthermore, differences between our study and prior studies with respect to study population, DNA extraction methodology, and variable region/s targeted may also limit comparability across studies. Of note, Mycoplasma penetrans, an organism previously associated with NGU among MSM (4), was not detected in our study. The V3-V4 primers used in our study have been shown to perform well *in silico* for detecting Mycoplasma species (49); therefore, it is possible that the absence of M. penetrans from our data set reflects a true low prevalence of this species in our study population.

In summary, our findings suggest that H. influenzae and specific Corynebacterium species may be etiological agents of idiopathic urethritis. Additionally, bacteria such as S. pneumoniae, S. pyogenes, E. coli, and Ureaplasma spp. that were found to dominate the urethral microbiota of cases and not controls may also account for some cases of urethritis and may have been the subject of case reports; however, larger studies are needed to elucidate the contribution of these organisms to the syndrome of NGU. Although our study was affected by a small sample size and reduced statistical power, the candidate organisms we have identified are biologically plausible etiologic agents of NGU that should be prioritized in future studies.

## MATERIALS AND METHODS

### Study design, patient population, and sample selection.

Participants and urine samples were derived from a case-control study conducted in 2004 to 2005 that examined viral and bacterial pathogens and behavioral practices associated with NGU (3). The parent study, which utilized nucleic acid amplification testing (NAAT) methods, found differences in the infectious causes of urethritis between MSM and MSW and found both adenoviruses and HSVs were significant causes of NGU. Men presenting to the Melbourne Sexual Health Centre (MSHC), Australia, between March 2004 and March 2005 were eligible. Cases in the parent study were men reporting urethral symptoms (discharge, dysuria, and urethral burning or irritation) who did not have urethral gonorrhea (i.e., absence of Gram-negative intracellular diplococci on urethral Gram stain and/or negative for N. gonorrhoeae on culture) or visible lesions consistent with genital herpes. Controls were men reporting no current urethral symptoms. All men completed a detailed questionnaire concerning urethral symptoms and sexual practices, underwent genital examination, and provided a first-pass urine specimen that was tested for the following organisms: C. trachomatis, M. genitalium, U. urealyticum, U. parvum, G. vaginalis, T. vaginalis, adenoviruses, and HSV-1 and HSV-2. Details of specific NAAT methods have been described (3). In the parent study, 64% of cases did not have an infectious cause of their symptoms identified, but this study was limited as only prespecified targets were examined.

The aim of the current study was to identify bacterial agents (not examined in the parent study) associated with urethritis in cases defined as having idiopathic urethritis. All men in the parent study who tested positive for C. trachomatis, M. genitalium, T. vaginalis, adenoviruses, HSV-1, and/or HSV-2 were excluded. Men with G. vaginalis, U. urealyticum, and/or U. parvum were not excluded because these organisms were not statistically associated with NGU in the parent study (3). The remaining 212 men with idiopathic urethritis (cases) were included in the microbiota study (Fig. 1). For this study, 212 controls were randomly selected from the parent study and frequency matched on self-report of a male sexual partner in the prior month to enrollment to ensure a similar proportion of MSM among cases and controls. Men who reported sex with a male partner in the month prior to enrollment were classified as MSM, and men who did not report sex with a male partner were classified as MSW. We did not match on age, as cases were significantly older than controls in the parent study (3), and we adjusted for age in analyses (see below).

### Laboratory methods.

DNA was extracted from stored urine samples using the PureLink Microbiome DNA purification kit and saliva and urine sample protocol (Invitrogen, publication MAN0014267), with the following variations: urine input volume was 1 mL and homogenization was performed by bead beating for 5 min at 50 Hz on the Tissue Lyzer (Qiagen). PCR amplification of the V3-V4 hypervariable regions of the 16S rRNA gene was performed using dual index universal primers (341F/805R), as previously described (50). Libraries were sequenced on the Illumina MiSeq platform using v3 chemistry (600-cycle kit; Illumina, San Diego, CA, USA) at Micromon Genomics (Monash University, Victoria, Australia). DNA extraction controls (phosphate-buffered saline [PBS]), PCR negative controls (ultrapure water) and positive controls (ZymoBIOMICS Microbial Community Standard, Zymo Research Corporation, Irvine, CA, USA) were extracted, processed as described, and sequenced alongside urine samples. Raw sequence reads are available from the NCBI Short Read Archive (Bioproject accession no. PRJNA831888).

### Sequence processing.

RStudio version 1.4.1717 (51) running R version 4.1.0 (52) was employed for all analyses and for generating figures. Demographics and sexual practices were compared using Fisher’s exact test for categorical variables and Wilcoxon rank-sum test for continuous variables.

The sequence data were processed as previously described (19), with some modifications. Adapters were removed using Cutadapt version 3.3 (53), and demultiplexing was performed using idemp (https://github.com/yhwu/idemp). Primers and heterogeneity spacers were removed using Cutadapt version 3.3 (53). DADA2 version 1.18.0 (54) was used for quality filtering, inferring amplicon sequence variants (ASVs), chimera identification, and merging of paired end reads. Kingdom to species level taxonomic assignment of ASVs was performed using DADA2 and the DADA2 formatted SILVA database version 138 (55). Species level assignment for key species (Lactobacillus, Streptococcus, Staphylococcus, and Haemophilus) was confirmed by a BLAST search against a database of 16S rRNA gene sequences from type strain organisms. Not all ASVs were able to be assigned to the species level.

Contamination from exogenous sources is a well described issue that can influence interpretation of resulting microbiota profiles (56) and is a particular issue for low-biomass samples, including urine. Therefore, we used a combination of methods to identify and remove potential contaminant ASVs. First, we applied the prevalence method in decontam version 1.12.0 (57) with a threshold of *P* = 0.25 and a sequencing run as a batch parameter. Second, we used SourceTracker2 version 2.0.1 (58) to estimate the proportion of source environments (positive and negative controls) present in each sink (urine sample) and the proportion of each ASV that was derived from each source environment. ASVs identified as a potential contaminant by decontam and/or SourceTracker2 were removed if they were not expected biologically. ASVs identified by decontam and SourceTracker2 as potential contaminants are provided in Table S1. Third, we removed ASVs that were of nonbacterial origin, that were not assigned at the phylum level, or that had a total abundance of <0.001%. Samples with ≥1,000 reads following contaminant filtering were selected for analyses. phyloseq version 1.38.0 (59) was used to store the ASV table, taxonomy, and metadata, and the resulting phyloseq object was used for all analyses.

10.1128/mbio.02213-22.1 TABLE S1 Amplicon sequence variants (ASVs) identified as contaminants by Decontam and SourceTracker2. Download Table S1, DOCX file, 0.02 MB. Copyright © 2022 Plummer et al. 2022 Plummer et al. https://creativecommons.org/licenses/by/4.0/ This content is distributed under the terms of the Creative Commons Attribution 4.0 International license.

### Statistics and data analysis.

Nonmetric multidimensional scaling (NMDS) and analysis of similarity (ANOSIM) were used to visualize and test for differences in the urethral microbiota composition between cases and controls, and between MSM and MSW. NMDS and ANOSIM were performed with vegan version 2.5.7 (60) using Bray-Curtis dissimilarities, and plots were drawn using ggplot2 version 3.3.5 (61). α-Diversity was calculated using the Shannon diversity index and was compared between cases and controls using linear regression, adjusting for sequence run and participant age.

ASVs with identical taxonomy were agglomerated using the microbiome package version 1.14.0 (62), and the agglomerated phyloseq object was used for all subsequent analyses. Heat maps were generated using ComplexHeatmap version 2.5.4 (63). The associated dendrograms were generated with vegan using hierarchical clustering of Bray-Curtis dissimilarities with Ward linkage.

Analysis of compositions of microbiomes with bias correction (ANCOM-BC) version 1.2.2 (64) was used to identify taxa that were differentially abundant between cases and controls. ANCOM-BC analysis was first conducted on the whole study population and then stratified by MSM status. ANCOM-BC analyses were also performed for each symptom (dysuria, urethral burning, and self-reported urethral discharge) and clinical sign (urethral discharge on examination and meatitis) to identify bacterial taxa associated with individual clinical characteristics, with stratification by MSM status. Additional ANCOM-BC analyses were performed to identify differences in the abundance of taxa by anatomical site of sexual exposure in the month prior to enrollment. ANCOM-BC analyses of sexual practices were conducted on the whole study population, and the following sexual practices were examined: receiving condomless oral sex, condomless vaginal sex, and insertive condomless anal sex. All ANCOM-BC analyses were adjusted for participant age and sequencing run, structural zeros were identified, and taxa present in ≤10% of samples were excluded. *P* values were corrected for multiple comparisons using the Benjamini-Hochberg method. Due to the truncated sample size and resulting lack of statistical power a false discovery rate (FDR)-corrected *P* value < 0.1 was considered significant.

### Study approval.

Ethical approval was obtained from the Human Research and Ethics Committee of the Alfred Hospital, Melbourne, Australia (approval 195/03). Written informed consent was obtained from all participants prior to any study-related procedures.
