# Supplementary figures and images for: The Urethral Microbiota of Men with and without Idiopathic Urethritis
Source: mBio. 2022 Oct 3;13(5):e02213-22. doi: 10.1128/mbio.02213-22 (PMC9600694; doi:10.1128/mbio.02213-22)

MSM

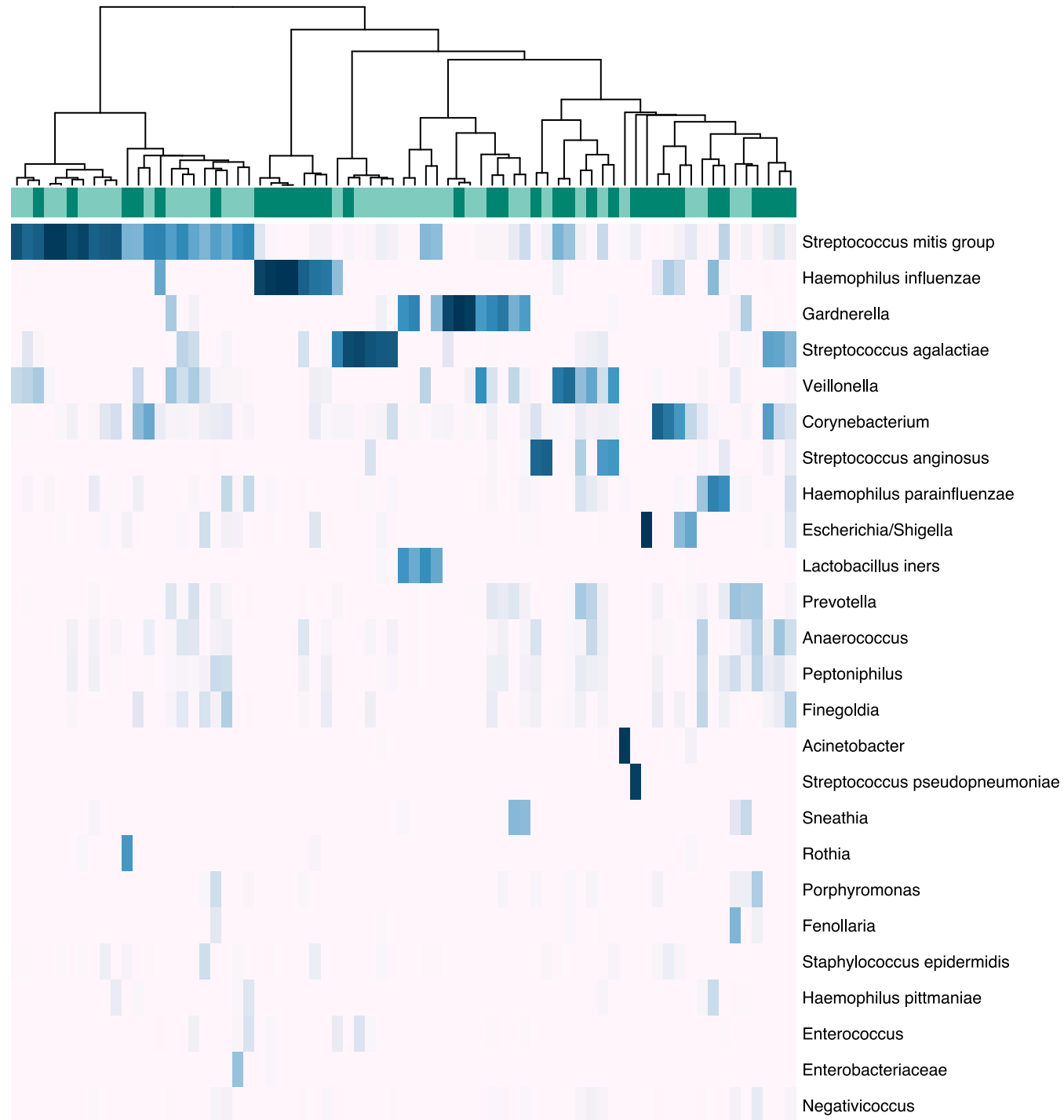

MSW

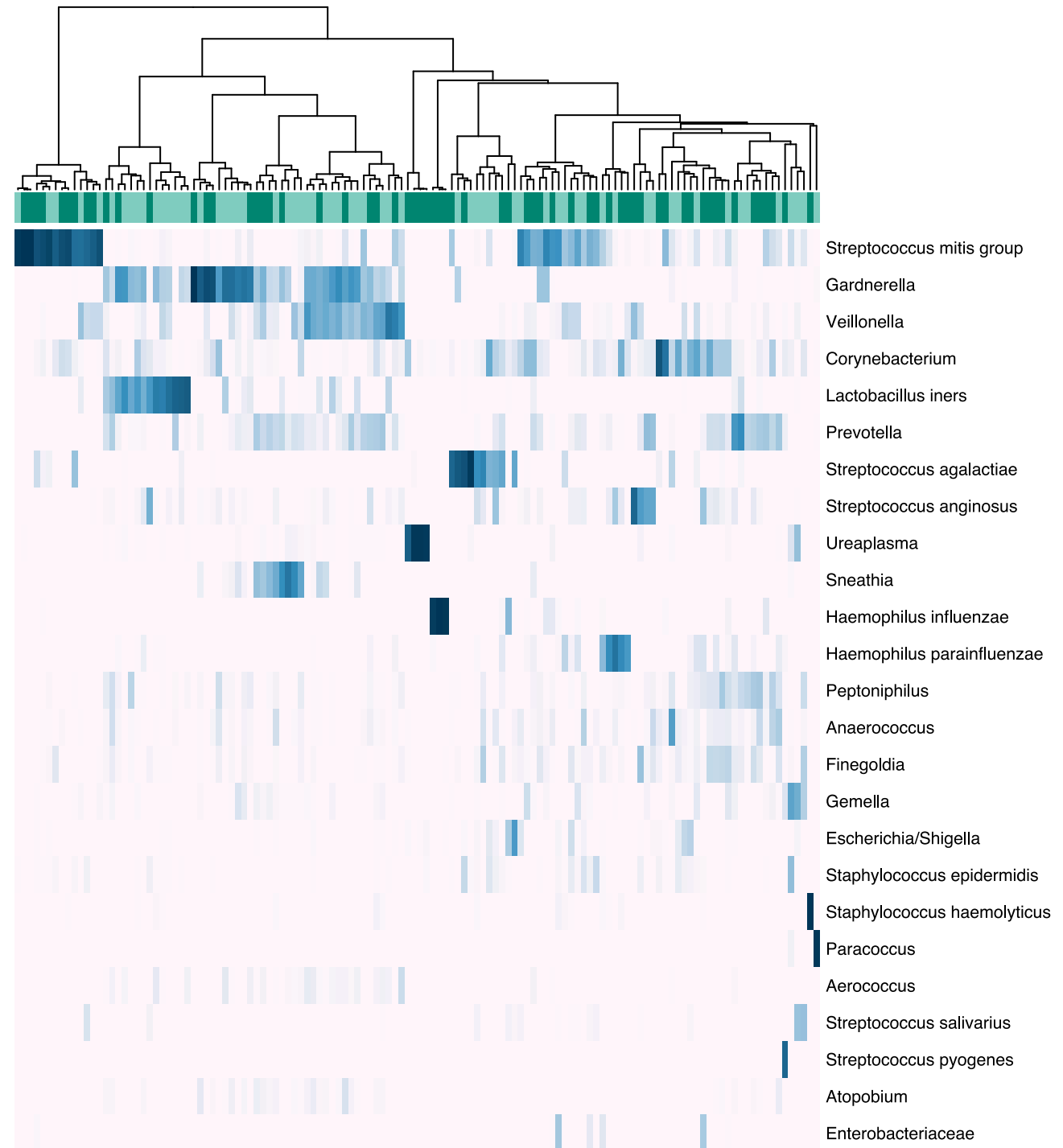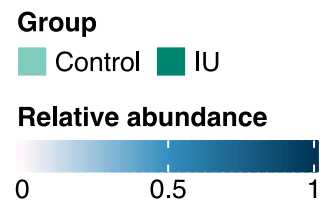

Supplement: FIG S1 [file mbio.02213-22-s0009.pdf]

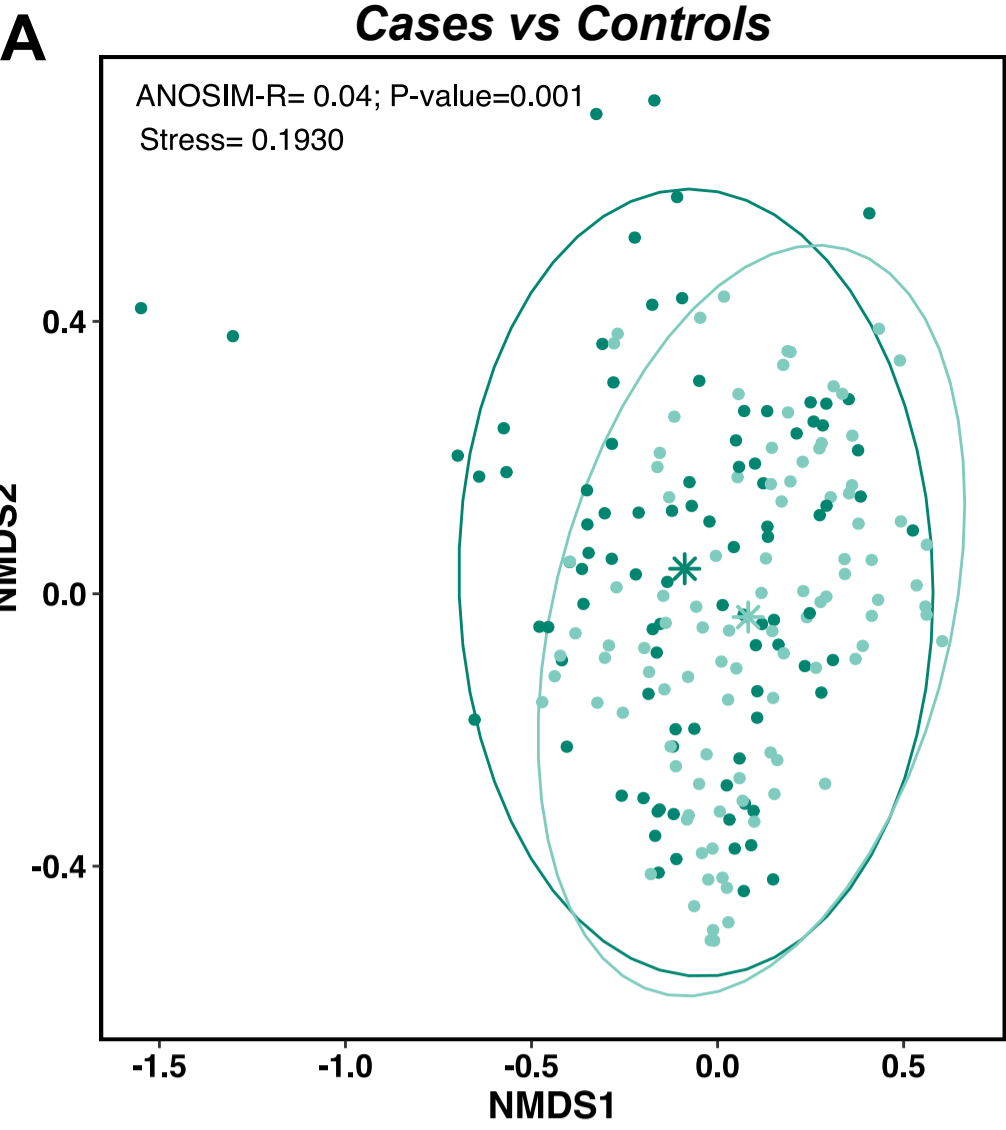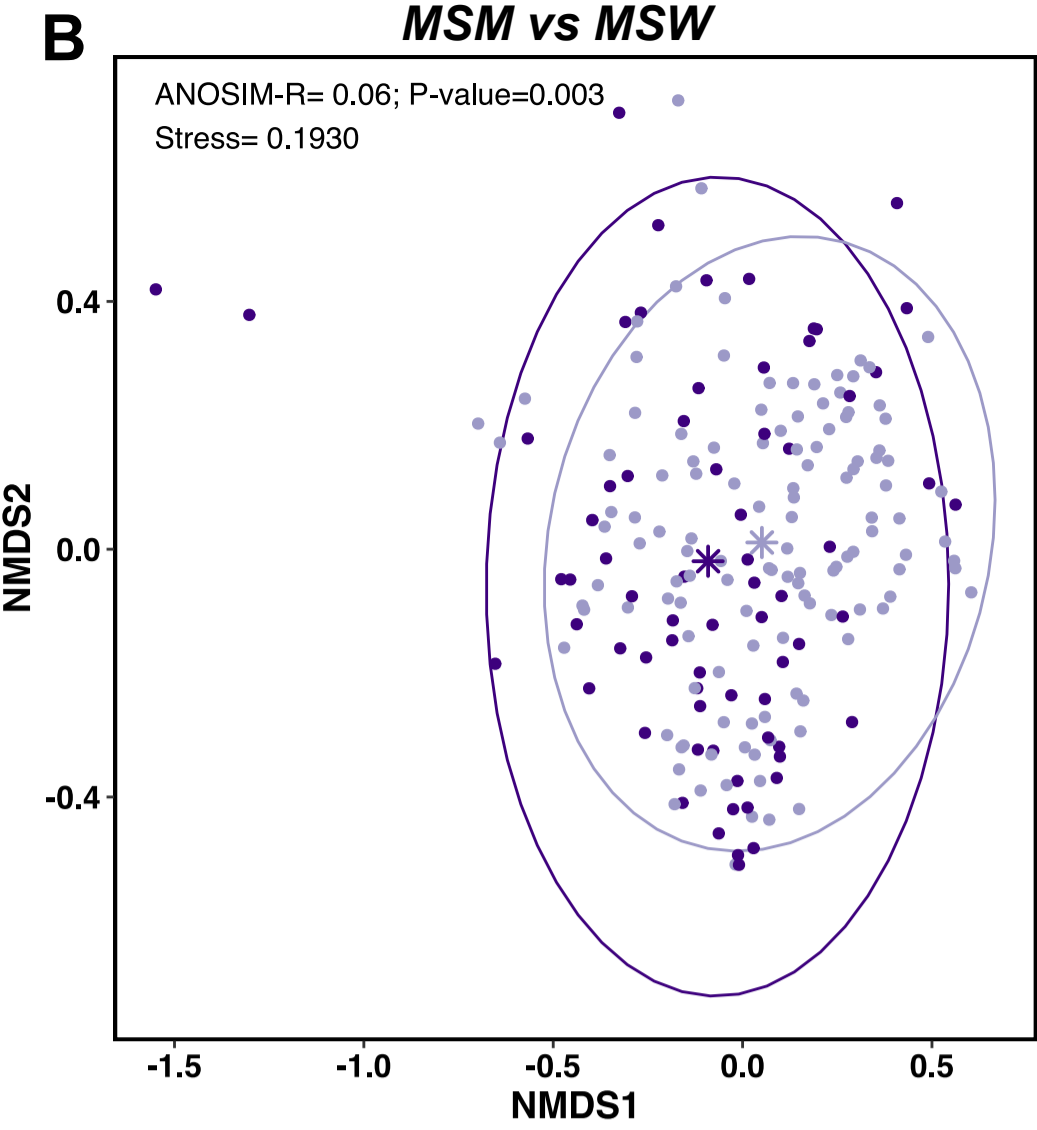

- IU cases
- Controls
- MSM
- MSW

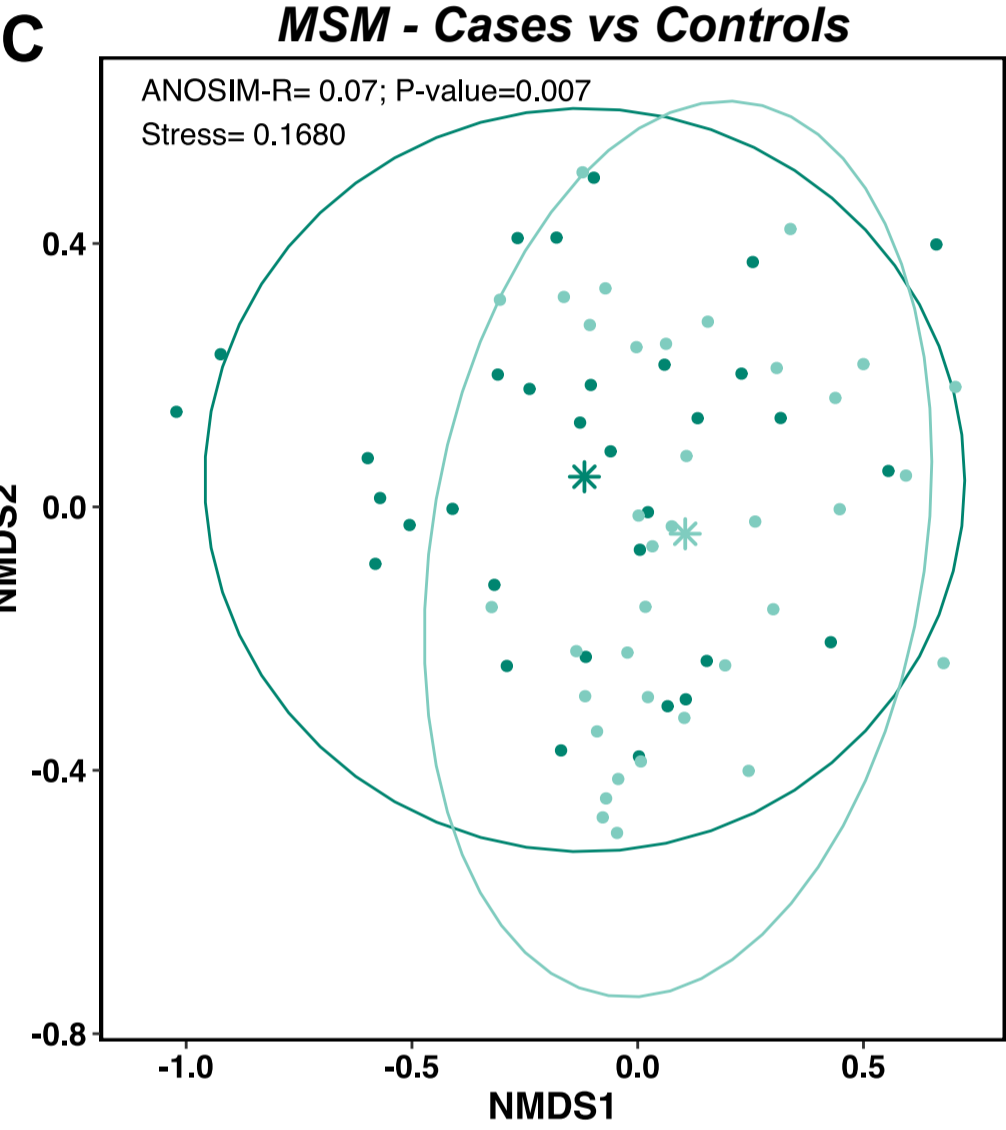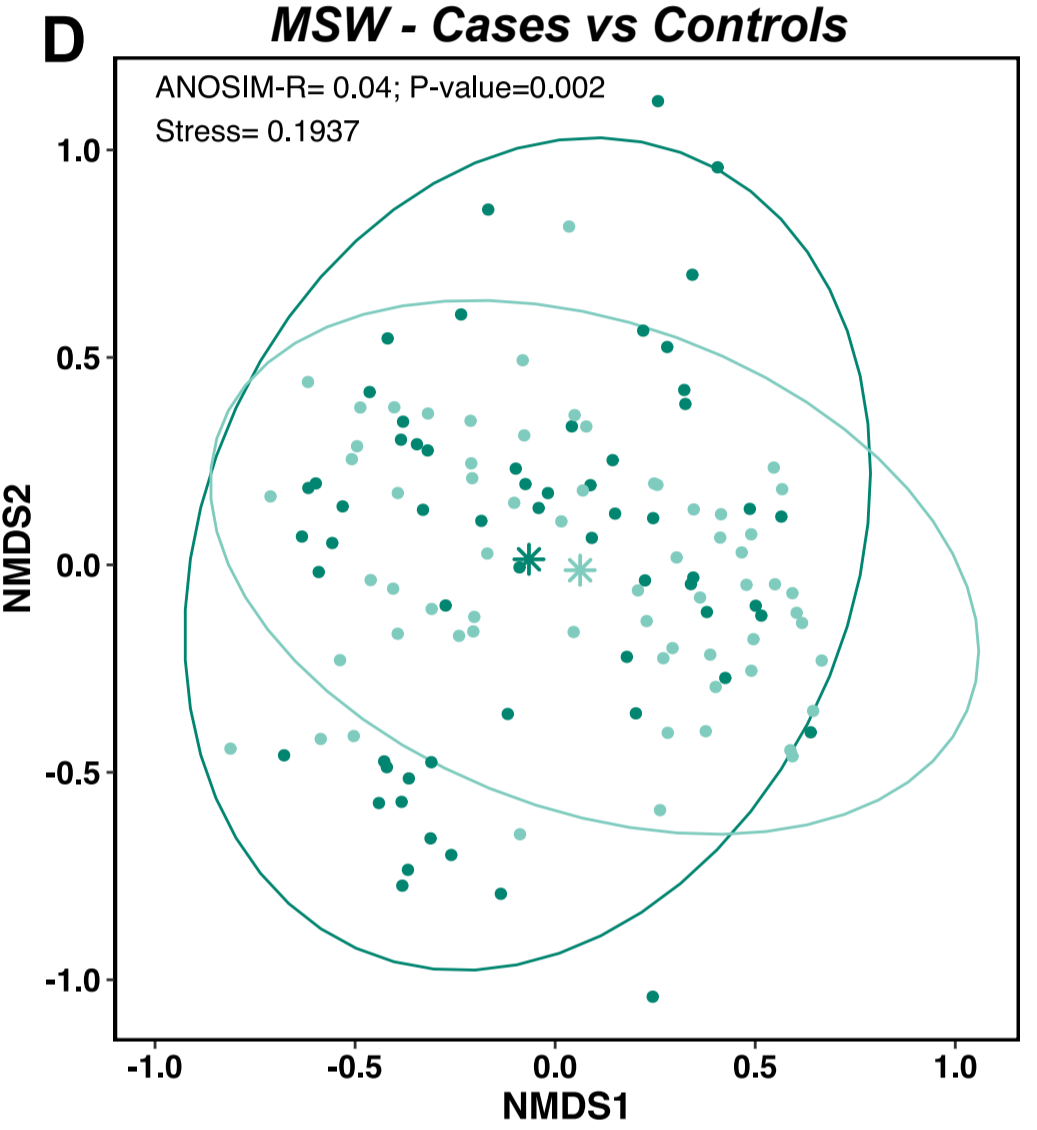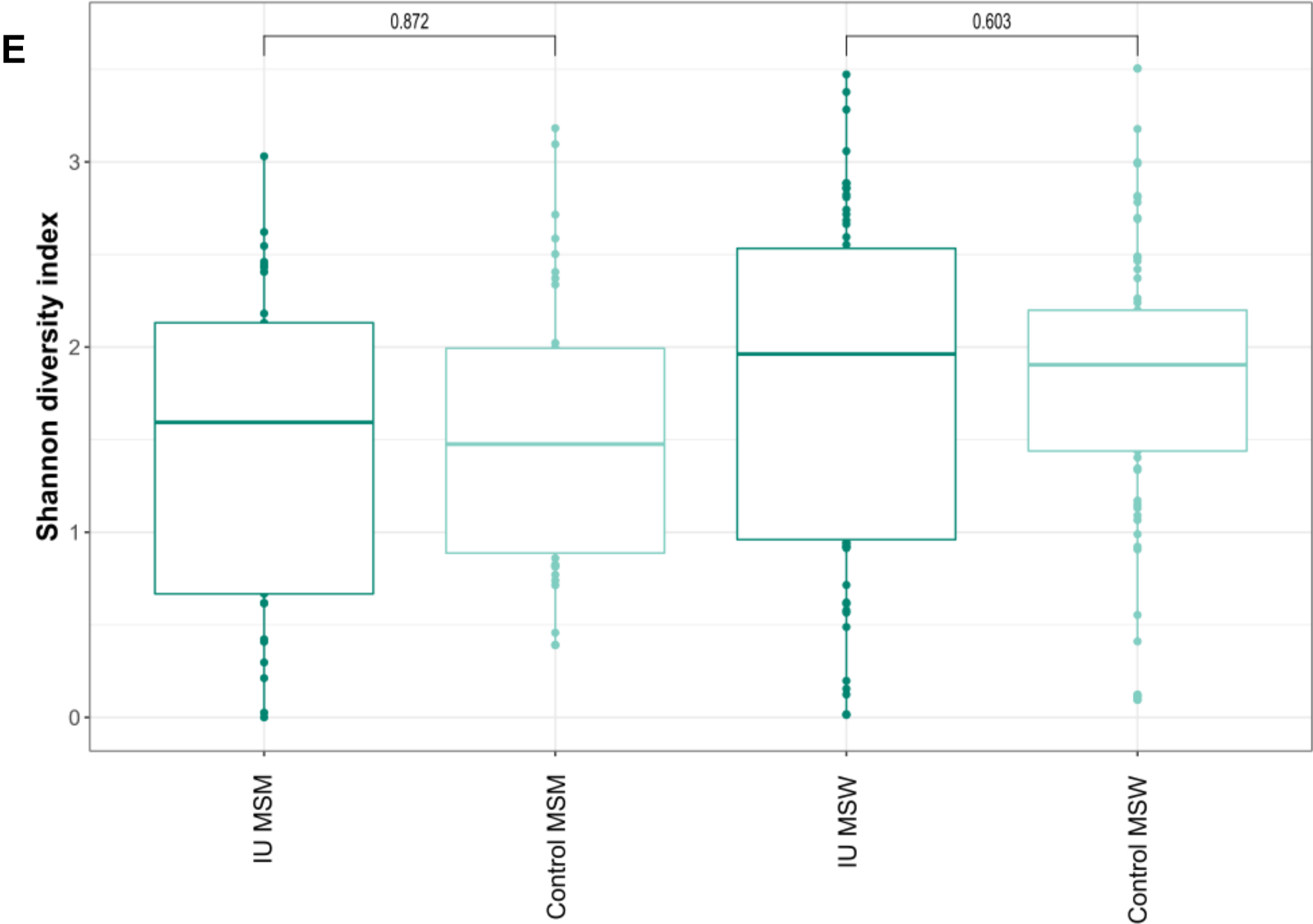

Supplement: FIG S2 [file mbio.02213-22-s0010.pdf]
