# Supplementary material for: The Urethral Microbiota of Men with and without Idiopathic Urethritis
Source: mBio. 2022 Oct 3;13(5):e02213-22. doi: 10.1128/mbio.02213-22 (PMC9600694; doi:10.1128/mbio.02213-22)
Supplement: TABLE S2 [file mbio.02213-22-s0002.docx]

**Table S2 – Sensitivity analysis exploring differentially abundant organisms between MSM with and without idiopathic urethritis**

|  | Idiopathic urethritis  n (%) | Control n (%) | Coeff.^b^ | Standard error | *P-*value | FDR adjusted  *P*-value |
| --- | --- | --- | --- | --- | --- | --- |
| **MSM^a^** | **N=30** | **N=36** |  |  |  |  |
| *Haemophilus influenzae* | 14 (47) | 4 (11) | 3.54 | 1.00 | <0.001 | **0.017** |
| *Porphyromonas* | 7 (23) | 7 (19) | 1.27 | 0.60 | 0.035 | 0.231 |
| *Haemophilus pittmaniae* | 2 (7) | 8 (22) | -0.95 | 0.41 | 0.021 | 0.231 |
| *L. iners* | 0 (0) | 7 (19) | -0.99 | 0.45 | NA | NA |
| *Gardnerella* | 6 (20) | 14 (39) | -1.79 | 0.83 | 0.031 | 0.231 |
| *Streptococcus mitis* group | 21 (70) | 32 (89) | -1.86 | 0.81 | 0.021 | 0.231 |

Abbreviations: Coeff., Coefficient; MSM, men who have sex with men

n = number of men with the specific taxon detected, % = n/N i.e. the percent of cases (or controls) with the specific taxon detected

Bold indicates that the difference was considered statistically significant (P < 0.05, FDR P < 0.1)

^a^ Five MSM (3 cases and 2 controls) who reported a female sexual partner in the month prior to enrolment were excluded from this sensitivity analysis.

^b^Coefficients were obtained from the ANCOM-BC log-linear (natural log) model. Positive coefficients indicate higher abundance in men with idiopathic urethritis, whereas negative coefficients indicate a higher abundance in control men. Analyses were adjusted for age and sequencing run, and only taxa with *P*<0.05 are included in this table
