# Supplementary material for: The Urethral Microbiota of Men with and without Idiopathic Urethritis
Source: mBio. 2022 Oct 3;13(5):e02213-22. doi: 10.1128/mbio.02213-22 (PMC9600694; doi:10.1128/mbio.02213-22)
Supplement: TABLE S3 [file mbio.02213-22-s0003.docx]

**Table S3 – Association of individual taxa with urethral burning**

|  | Burning n (%) | No burning n (%) | Coeff.^a^ | Standard error | *P-*value | FDR adjusted  *P*-value |
| --- | --- | --- | --- | --- | --- | --- |
| **MSM** | **N=23** | **N=48** |  |  |  |  |
| *Haemophilus influenzae* | 12 (52) | 7 (15) | 2.83 | 0.87 | 0.001 | **0.039** |
| *Enterococcus* | 2 (9) | 10 (21) | -0.82 | 0.34 | 0.017 | 0.182 |
| *Veillonella* | 10 (43) | 21 (53) | -1.36 | 0.68 | 0.044 | 0.281 |
| *Streptococcus mitis group* | 15 (65) | 42 (88) | -1.62 | 0.77 | 0.035 | 0.281 |
| *Gardnerella* | 2 (9) | 18 (38) | -2.06 | 0.73 | 0.005 | **0.079** |
| **MSW** | **N=40** | **N=88** |  |  |  |  |
| *Corynebacterium* | 36 (90) | 76 (86) | 0.92 | 0.41 | 0.024 | 0.213 |
| *Staphylococcus hominis/xylosus* | 10 (25) | 11 (13) | 0.76 | 0.32 | 0.019 | 0.209 |
| *Negativicoccus* | 5 (13) | 33 (38) | -0.84 | 0.26 | 0.001 | **0.067** |
| *Aerococcus* | 6 (15) | 28 (32) | -0.85 | 0.34 | 0.011 | 0.169 |
| *Prevotella* | 16 (40) | 60 (68) | -1.31 | 0.50 | 0.009 | 0.169 |

Abbreviations: Coeff., Coefficient; MSM, men who have sex with men; MSW, men who have sex with women.

n = number of men with the specific taxon detected, % = n/N

Bold indicates that the difference was considered statistically significant (P < 0.05, FDR P < 0.1)

^a^ Coefficients were obtained from the ANCOM-BC log-linear (natural log) model. Positive coefficients indicate higher abundance in men with urethral burning, whereas negative coefficients indicate a higher abundance in men without urethral burning. Analyses were adjusted for age and sequencing run, and only taxa with *P*<0.05 are included in this table
