# Supplementary material for: The Urethral Microbiota of Men with and without Idiopathic Urethritis
Source: mBio. 2022 Oct 3;13(5):e02213-22. doi: 10.1128/mbio.02213-22 (PMC9600694; doi:10.1128/mbio.02213-22)
Supplement: TABLE S4 [file mbio.02213-22-s0004.docx]

**Table S4 – Association of individual taxa with dysuria**

|  | Dysuria n (%) | No dysuria n (%) | Coeff.^a^ | Standard error | *P-*value | FDR adjusted  *P*-value |
| --- | --- | --- | --- | --- | --- | --- |
| **MSM** | **N=23** | **N=48** |  |  |  |  |
| *Haemophilus influenzae* | 11 (49) | 8 (17) | 3.44 | 0.95 | <0.001 | **0.010** |
| *Atopobium ^b^* | 0 (0) | 8 (17) | -0.11 | 0.23 | NA | NA |
| *Gardnerella* | 4 (17) | 16 (33) | -1.60 | 0.69 | 0.020 | 0.325 |
| **MSW** | **N=41** | **N=87** |  |  |  |  |
| *Atopobium* | 6 (15) | 27 (31) | -0.72 | 0.31 | 0.022 | 0.198 |
| *Gemella* | 11 (27) | 41 (47) | -0.83 | 0.38 | 0.029 | 0.220 |
| *Negativicoccus* | 5 (12) | 33 (38) | -0.83 | 0.26 | 0.001 | **0.065** |
| *Aerococcus* | 6 (15) | 28 (32) | -0.89 | 0.33 | 0.006 | **0.082** |
| *Finegoldia* | 19 (46) | 64 (74) | -1.05 | 0.37 | 0.005 | **0.082** |
| *Prevotella* | 17 (41) | 59 (68) | -1.34 | 0.50 | 0.007 | **0.082** |

Abbreviations: Coeff., Coefficient; MSM, men who have sex with men; MSW, men who have sex with women

n = number of men with the specific taxon detected, % = n/N

Bold indicates that the difference was considered statistically significant (P < 0.05, FDR P < 0.1)

^a^ Coefficients were obtained from the ANCOM-BC log-linear (natural log) model. Positive coefficients indicate higher abundance in men with dysuria, whereas negative coefficients indicate a higher abundance in men without dysuria. Analyses were adjusted for age and sequencing run, and only those taxa with *P*<0.05 are included in this table

^b^ Taxon identified as a structural zero (i.e. present in one group but absent, or close to absent, from the comparator). Taxa identified as structural zeros are excluded from analyses and thus do not have a corresponding p-value.
