# Supplementary material for: The Urethral Microbiota of Men with and without Idiopathic Urethritis
Source: mBio. 2022 Oct 3;13(5):e02213-22. doi: 10.1128/mbio.02213-22 (PMC9600694; doi:10.1128/mbio.02213-22)
Supplement: TABLE S5 [file mbio.02213-22-s0005.docx]

**Table S5 – Association of individual taxa with self-reported urethral discharge**

|  | Self-reported urethral discharge n (%) | No self-reported urethral discharge n (%) | Coeff.^a^ | Standard error | *P-*value | FDR adjusted  *P*-value |
| --- | --- | --- | --- | --- | --- | --- |
| **MSM** | **N=23** | **N=48** |  |  |  |  |
| *Haemophilus influenzae* | 13 (57) | 6 (13) | 4.00 | 0.94 | <0.001 | **<0.001** |
| *Porphyromonas* | 6 (26) | 9 (19) | 1.22 | 0.55 | 0.027 | 0.215 |
| *Staphylococcus haemolyticus ^b^* | 0 (0) | 9 (19) | -0.59 | 0.29 | NA | NA |
| *Veillonella* | 9 (39) | 29 (60) | -1.81 | 0.59 | 0.002 | **0.033** |
| *Streptococcus mitis group* | 16 (70) | 41 (85) | -2.07 | 0.75 | 0.006 | **0.066** |
| **MSW** | **N=34** | **N=94** |  |  |  |  |
| *Haemophilus influenzae* | 6 (18) | 14 (15) | 1.14 | 0.52 | 0.028 | 0.139 |
| *Lawsonella* | 7 (21) | 13 (14) | 0.96 | 0.25 | <0.001 | **0.003** |
| *Staphylococcus haemolyticus* | 5 (15) | 13 (14) | 0.83 | 0.41 | 0.044 | 0.179 |
| *Cutibacterium* | 3 (9) | 11 (12) | 0.74 | 0.21 | <0.001 | **0.005** |
| *Facklamia* | 4 (12) | 9 (10) | 0.67 | 0.21 | 0.001 | **0.013** |
| *Streptococcus cristatus* | 5 (15) | 19 (20) | 0.62 | 0.18 | 0.001 | **0.008** |
| *Staphylococcus hominis/xylosus* | 6 (18) | 15 (16) | 0.57 | 0.28 | 0.042 | 0.179 |
| *Varibaculum* | 3 (9) | 11 (12) | 0.54 | 0.18 | 0.004 | **0.026** |
| *Howardella* | 2 (6) | 12 (13) | 0.34 | 0.14 | 0.012 | **0.076** |
| *Lactobacillus iners* | 4 (12) | 36 (38) | -1.14 | 0.47 | 0.016 | **0.088** |
| *Aerococcus ^b^* | 0 (0) | 34 (36) | -1.17 | 0.26 | NA | NA |

Abbreviations: Coeff., Coefficient; MSM, men who have sex with men; MSW, men who have sex with women

n = number of men with the specific taxon detected, % = n/N

Bold indicates that the difference was considered statistically significant (P < 0.05, FDR P < 0.1)

^a^ Coefficients were obtained from the ANCOM-BC log-linear (natural log) model. Positive coefficients indicate higher abundance in men with self-reported urethral discharge, whereas negative coefficients indicate a higher abundance in men without self-reported urethral discharge. Analyses were adjusted for age and sequencing run, and only taxa with *P*<0.05 are included in this table.

^b^ Taxon identified as a structural zero (i.e. present in one group but absent, or close to absent, from the comparator). Taxa identified as structural zeros are excluded from analyses and thus do not have a corresponding p-value.
