# Supplementary material for: The Urethral Microbiota of Men with and without Idiopathic Urethritis
Source: mBio. 2022 Oct 3;13(5):e02213-22. doi: 10.1128/mbio.02213-22 (PMC9600694; doi:10.1128/mbio.02213-22)
Supplement: TABLE S6 [file mbio.02213-22-s0006.docx]

**Table S6 – Association of individual taxa with urethral discharge on examination**

|  | Urethral discharge on examination n (%) | No urethral discharge on examination n (%) | Coeff.^a^ | Standard error | *P-*value | FDR adjusted  *P*-value |
| --- | --- | --- | --- | --- | --- | --- |
| **MSM** | **N=19** | **N=52** |  |  |  |  |
| *Haemophilus influenzae* | 10 (52) | 9 (17) | 2.92 | 1.04 | 0.005 | **0.065** |
| *Staphylococcus haemolyticus ^b^* | 0 (0) | 9 (17) | -0.70 | 0.31 | NA | NA |
| *Haemophilus pittmaniae ^b^* | 0 (0) | 10 (19) | -0.92 | 0.34 | NA | NA |
| *Streptococcus mitis group* | 13 (68) | 44 (85) | -1.89 | 0.85 | 0.026 | 0.168 |
| Veillonella | 7 (37) | 31 (60) | -2.04 | 0.58 | <0.001 | **0.015** |
| **MSW** | **N=28** | **N=100** |  |  |  |  |
| *Corynebacterium* | 26 (93) | 86 (86) | 0.92 | 0.42 | 0.027 | 0.102 |
| *Lawsonella* | 8 (29) | 12 (12) | 0.81 | 0.28 | 0.004 | **0.025** |
| *Staphylococcus hominis/xylosus* | 8 (29) | 13 (13) | 0.71 | 0.33 | 0.032 | 0.109 |
| *Coriobacteriales bacterium DNF00809* | 2 (7) | 25 (25) | -0.61 | 0.24 | 0.010 | **0.045** |
| *Parvimonas ^b^* | 0 (0) | 21 (21) | -0.71 | 0.21 | NA | NA |
| *Atopobium* | 2 (7) | 31 (31) | -1.08 | 0.24 | <0.001 | **<0.001** |
| *Gemella* | 5 (18) | 47 (47) | -1.11 | 0.39 | 0.004 | **0.025** |
| *Sneathia ^b^* | 0 (0) | 20 (20) | -1.16 | 0.31 | NA | NA |
| *Prevotella* | 13 (46) | 63 (63) | -1.22 | 0.51 | 0.018 | **0.073** |
| *Aerococcus ^b^* | 0 (0) | 34 (34) | -1.43 | 0.25 | NA | NA |
| *Veillonella* | 11 (39) | 55 (55) | -1.44 | 0.55 | 0.009 | **0.045** |
| *Lactobacillus iners* | 2 (7) | 38 (38) | -1.77 | 0.44 | 0.000 | **0.001** |
| *Gardnerella* | 7 (25) | 53 (53) | -1.92 | 0.71 | 0.007 | **0.040** |

Abbreviations: Coeff., Coefficient; MSM, men who have sex with men; MSW, men who have sex with women

n = number of men with the specific taxon detected, % = n/N

Bold indicates that the difference was considered statistically significant (P < 0.05, FDR P < 0.1)

^a^ Coefficients were obtained from the ANCOM-BC log-linear (natural log) model. Positive coefficients indicate higher abundance in men with urethral discharge on examination, whereas negative coefficients indicate a higher abundance in men without urethral discharge on examination. Analyses were adjusted for age and sequencing run, and only taxa with *P*<0.05 are included in this table

^b^ Taxon identified as a structural zero (i.e. present in one group but absent, or close to absent, from the comparator). Taxa identified as structural zeros are excluded from analyses and thus do not have a corresponding p-value.
