# Supplementary material for: The Urethral Microbiota of Men with and without Idiopathic Urethritis
Source: mBio. 2022 Oct 3;13(5):e02213-22. doi: 10.1128/mbio.02213-22 (PMC9600694; doi:10.1128/mbio.02213-22)
Supplement: TABLE S7 [file mbio.02213-22-s0007.docx]

**Table S7 – Association of individual taxa with meatitis**

|  | Meatitis n (%) | No meatitis n (%) | Coeff.^a^ | Standard error | *P-*value | FDR adjusted  *P*-value |
| --- | --- | --- | --- | --- | --- | --- |
| **MSM** | N=16 | N=55 |  |  |  |  |
| *Haemophilus influenzae* | 7 (44) | 12 (22) | 2.27 | 1.07 | 0.034 | 0.219 |
| *Staphylococcus haemolyticus ^b^* | 0 (0) | 9 (16) | -0.53 | 0.31 | NA | NA |
| *Enterococcus* | 1 (6) | 11 (20) | -0.67 | 0.34 | 0.046 | 0.246 |
| *Haemophilus pittmaniae ^b^* | 0 (0) | 10 (18) | -0.77 | 0.36 | NA | NA |
| *Haemophilus parainfluenzae* | 4 (25) | 27 (49) | -1.48 | 0.56 | 0.008 | 0.162 |
| *Veillonella* | 6 (38) | 32 (58) | -1.49 | 0.62 | 0.015 | 0.162 |
| *Streptococcus mitis* group | 9 (56) | 48 (87) | -2.23 | 0.91 | 0.014 | 0.162 |
| **MSW** | N=19 | N=108 |  |  |  |  |
| *Cutibacterium ^b^* | 1 (5) | 13 (12) | -0.55 | 0.21 | NA | NA |
| *Acinetobacter* | 2 (11) | 15 (13) | -0.59 | 0.28 | 0.036 | 0.229 |
| *Facklamia ^b^* | 0 (0) | 13 (12) | -0.63 | 0.22 | NA | NA |
| *Micrococcus* | 2 (11) | 17 (16) | -0.65 | 0.26 | 0.011 | 0.101 |
| *Parvimonas* | 1 (5) | 20 (19) | -0.69 | 0.32 | 0.029 | 0.218 |
| *Haemophilus influenzae* | 2 (11) | 18 (17) | -0.78 | 0.39 | 0.048 | 0.233 |
| *Streptococcus salivarius ^b^* | 1 (5) | 17 (16) | -0.87 | 0.25 | NA | NA |
| *Escherichia/Shigella* | 3 (16) | 36 (33) | -0.99 | 0.36 | 0.006 | **0.095** |
| *Staphylococcus epidermidis* | 5 (26) | 50 (46) | -1.12 | 0.55 | 0.044 | 0.233 |

Abbreviations: Coeff., Coefficient; MSM, men who have sex with men;

n = number of men with the specific taxon detected, % = n/N

Bold indicates that the difference was considered statistically significant (P < 0.05, FDR P < 0.1)

^a^ Coefficients were obtained from the ANCOM-BC log-linear (natural log) model. Positive coefficients indicate higher abundance in men with meatitis, whereas negative coefficients indicate a higher abundance in men without meatitis. Analyses were adjusted for age and sequencing run, and only taxa with *P*<0.05 are included in this table

^b^ Taxon identified as a structural zero (i.e. present in one group but absent, or close to absent, from the comparator). Taxa identified as structural zeros are excluded from analyses and thus do not have a corresponding p-value.
