# Supplementary material for: The Urethral Microbiota of Men with and without Idiopathic Urethritis
Source: mBio. 2022 Oct 3;13(5):e02213-22. doi: 10.1128/mbio.02213-22 (PMC9600694; doi:10.1128/mbio.02213-22)
Supplement: TABLE S8 [file mbio.02213-22-s0008.docx]

**Table S8 – Association of individual taxa with sexual exposure**

|  | **Oral sex**  **N=175**  **n (%)** | **No oral sex**  **N=22**  **n (%)** | **Coeff.^a^** | **Standard error** | ***P-*value** | **FDR adjusted**  ***P*-value** |
| --- | --- | --- | --- | --- | --- | --- |
| *Streptococcus mitis* group | 123 (70) | 12 (55) | 1.66 | 0.71 | 0.019 | 0.117 |
| *Streptococcus agalactiae* | 51 (29) | 2 (9) | 1.42 | 0.54 | 0.009 | 0.117 |
| *Haemophilus influenzae* | 35 (20) | 3 (14) | 0.97 | 0.50 | 0.054 | 0.259 |
| *Ureaplasma* | 50 (29) | 4 (18) | 0.81 | 0.29 | 0.005 | 0.117 |
| *Facklamia* | 20 (11) | 1 (5) | 0.63 | 0.25 | 0.012 | 0.117 |
| *Coriobacteriales bacterium DNF00809* | 28 (16) | 3 (14) | 0.50 | 0.24 | 0.037 | 0.199 |
| *Streptococcus cristatus* | 35 (20) | 3 (14) | 0.47 | 0.20 | 0.019 | 0.117 |
| *Finegoldia* | 100 (57) | 17 (77) | -0.94 | 0.39 | 0.017 | 0.117 |
| *Corynebacterium* | 148 (85) | 22 (100) | -1.04 | 0.41 | 0.011 | 0.117 |
|  | **Vaginal sex**  **N=98**  **n (%)** | **No vaginal sex**  **N=96**  **n (%)** | **Coeff.^a^** | **Standard error** | ***P-*value** | **FDR adjusted**  ***P*-value** |
| *Lactobacillus iners* | 32 (33) | 13 (14) | 1.25 | 0.40 | 0.002 | **0.017** |
| *Prevotella* | 58 (59) | 44 (46) | 1.05 | 0.37 | 0.004 | **0.037** |
| *Ureaplasma* | 40 (41) | 14 (15) | 1.03 | 0.29 | 0.000 | **0.017** |
| *Gardnerella* | 46 (47) | 31 (32) | 1.01 | 0.50 | 0.043 | 0.187 |
| *Streptococcus anginosus* | 47 (48) | 28 (29) | 0.77 | 0.36 | 0.034 | 0.161 |
| *Atopobium* | 27 (28) | 13 (14) | 0.77 | 0.24 | 0.001 | **0.017** |
| *Staphylococcus epidermidis* | 46 (47) | 30 (31) | 0.73 | 0.31 | 0.018 | 0.109 |
| *Aerococcus* | 26 (27) | 12 (13) | 0.70 | 0.26 | 0.006 | **0.044** |
| *Coriobacteriales bacterium DNF00809* | 23 (23) | 7 (7) | 0.68 | 0.20 | 0.001 | **0.017** |
| *Streptococcus mitis* group | 61 (62) | 72 (75) | -1.06 | 0.48 | 0.027 | 0.143 |
|  | **Anal sex**  **N=47**  **n (%)** | **No anal sex**  **N=149**  **n (%)** | **Coeff.^a^** | **Standard error** | ***P-*value** | **FDR adjusted**  ***P*-value** |
| *Haemophilus parainfluenzae* | 25 (53) | 40 (27) | 1.13 | 0.42 | 0.007 | 0.151 |
| *Coriobacteriales bacterium DNF00809* | 4 (9) | 26 (17) | -0.56 | 0.20 | 0.004 | 0.151 |
| *Haemophilus influenzae* | 8 (17) | 31 (21) | -0.70 | 0.31 | 0.027 | 0.383 |

Data missing for up to n=5 participants; Abbreviations: Coeff., Coefficient;

n = number of men with the specific taxon detected, % = n/N

Bold indicates that the difference was considered statistically significant (P < 0.05, FDR P < 0.1)

^a^Coefficients were obtained from the ANCOM-BC log-linear (natural log) model. Positive coefficients indicate higher abundance in men reporting the specific sexual exposure, whereas negative coefficients indicate a higher abundance in men who did not report the exposure. Analyses were adjusted for age and sequencing run, and only taxa with *P*<0.05 are included in this table
