# Supplementary material for: The Urethral Microbiota of Men with and without Idiopathic Urethritis
Source: mBio. 2022 Oct 3;13(5):e02213-22. doi: 10.1128/mbio.02213-22 (PMC9600694; doi:10.1128/mbio.02213-22)
Supplement: TABLE S1 [file mbio.02213-22-s0001.docx]

# **Table S1 - ASVs identified as contaminant by Decontam and SourceTracker2**

| **Phylum** | **Class** | **Order** | **Family** | **Genus** | **Contaminant identified by Decontam** | **Contaminant identified by SourceTracker** | **ASV removed as potential contaminant** |
| --- | --- | --- | --- | --- | --- | --- | --- |
| Proteobacteria | Gammaproteobacteria | Burkholderiales | Burkholderiaceae | Burkholderia-Caballeronia-Paraburkholderia | 1 | 1 | 1 |
| Proteobacteria | Alphaproteobacteria | Rhizobiales | Rhizobiaceae | Allorhizobium-Neorhizobium-Pararhizobium-Rhizobium | 1 | 1 | 1 |
| Proteobacteria | Gammaproteobacteria | Burkholderiales | Burkholderiaceae | Burkholderia-Caballeronia-Paraburkholderia | 1 | 1 | 1 |
| Actinobacteriota | Actinobacteria | Micrococcales | Microbacteriaceae | Leifsonia | 1 | 1 | 1 |
| Proteobacteria | Gammaproteobacteria | Burkholderiales | Burkholderiaceae | Burkholderia-Caballeronia-Paraburkholderia | 1 | 1 | 1 |
| Cyanobacteria | Cyanobacteriia | Chloroplast | NA | NA | 1 | 0 | 1 |
| Firmicutes | Bacilli | Lactobacillales | Leuconostocaceae | Weissella | 1 | 0 | 1 |
| Proteobacteria | Gammaproteobacteria | Burkholderiales | Burkholderiaceae | Burkholderia-Caballeronia-Paraburkholderia | 1 | 0 | 1 |
| Firmicutes | Bacilli | Lactobacillales | Lactobacillaceae | Lactobacillus | 1 | 0 | 0 |
| Firmicutes | Bacilli | Lactobacillales | Streptococcaceae | Streptococcus | 1 | 0 | 0 |
| Firmicutes | Bacilli | Lactobacillales | Streptococcaceae | Streptococcus | 1 | 0 | 0 |
| Proteobacteria | Gammaproteobacteria | Pasteurellales | Pasteurellaceae | Haemophilus | 1 | 0 | 0 |
| Firmicutes | Bacilli | Staphylococcales | Staphylococcaceae | Staphylococcus | 1 | 0 | 0 |
| Proteobacteria | Gammaproteobacteria | Burkholderiales | Burkholderiaceae | Burkholderia-Caballeronia-Paraburkholderia | 0 | 1 | 1 |
| Proteobacteria | Gammaproteobacteria | Burkholderiales | Comamonadaceae | Pelomonas | 0 | 1 | 1 |
| Bacteroidota | Bacteroidia | Flavobacteriales | Weeksellaceae | Empedobacter | 0 | 1 | 1 |
| Proteobacteria | Gammaproteobacteria | Burkholderiales | Burkholderiaceae | Burkholderia-Caballeronia-Paraburkholderia | 0 | 1 | 1 |
| Proteobacteria | Gammaproteobacteria | Burkholderiales | Burkholderiaceae | Burkholderia-Caballeronia-Paraburkholderia | 0 | 1 | 1 |
| Proteobacteria | Alphaproteobacteria | Acetobacterales | Acetobacteraceae | Roseomonas | 0 | 1 | 1 |
| Proteobacteria | Gammaproteobacteria | Burkholderiales | Burkholderiaceae | NA | 0 | 1 | 1 |
| Proteobacteria | Gammaproteobacteria | Burkholderiales | NA | NA | 0 | 1 | 1 |
| Proteobacteria | Gammaproteobacteria | Pseudomonadales | Moraxellaceae | Acinetobacter | 0 | 1 | 1 |
| Proteobacteria | Gammaproteobacteria | Burkholderiales | NA | NA | 0 | 1 | 1 |
| Proteobacteria | Gammaproteobacteria | Burkholderiales | Burkholderiaceae | Burkholderia-Caballeronia-Paraburkholderia | 0 | 1 | 1 |
| Proteobacteria | Gammaproteobacteria | Burkholderiales | Burkholderiaceae | NA | 0 | 1 | 1 |
| Proteobacteria | Gammaproteobacteria | Burkholderiales | NA | NA | 0 | 1 | 1 |
| Fusobacteriota | Fusobacteriia | Fusobacteriales | Leptotrichiaceae | Leptotrichia | 0 | 1 | 1 |
| Firmicutes | Bacilli | Paenibacillales | Paenibacillaceae | Paenibacillus | 0 | 1 | 1 |
| Proteobacteria | Alphaproteobacteria | Rhizobiales | Rhizobiaceae | Allorhizobium-Neorhizobium-Pararhizobium-Rhizobium | 0 | 1 | 1 |
| Proteobacteria | Gammaproteobacteria | Burkholderiales | Burkholderiaceae | Burkholderia-Caballeronia-Paraburkholderia | 0 | 1 | 1 |
| Firmicutes | Bacilli | Bacillales | Marinococcaceae | NA | 0 | 1 | 1 |
| Firmicutes | Bacilli | Lactobacillales | Streptococcaceae | Streptococcus | 0 | 1 | 1 |

ASV, amplicon sequence variant
